# Examination of human osteoarchaeological remains as a feasible source of polar and apolar metabolites to study past conditions

**DOI:** 10.1038/s41598-023-27401-0

**Published:** 2023-01-13

**Authors:** Diego Badillo-Sanchez, Maria Serrano Ruber, Anna M. Davies-Barrett, Jatinderpal K. Sandhu, Donald J. L. Jones, Martin Hansen, Sarah A. Inskip

**Affiliations:** 1 School of Archaeology and Ancient History, University of Leicester, Leicester, UK; 2 Leicester Cancer Research Centre, RKCSB, University of Leicester, Leicester, UK; 3 The Leicester Van Geest MultiOmics Facility, University of Leicester, Leicester, UK; 4 Environmental Metabolomics Lab, Dept. of Environmental Science, Aarhus University, Aarhus, Denmark

**Keywords:** Biochemistry, Biomarkers

## Abstract

Metabolomics is a modern tool that aids in our understanding of the molecular changes in organisms. Archaeological science is a branch of archaeology that explores different archaeological materials using modern analytical tools. Human osteoarchaeological material are a frequent finding in archaeological contexts and have the potential to offer information about previous human populations, which can be illuminating about our current condition. Using a set of samples comprising different skeletal elements and bone structures, here we explore for the first time the possibility of extracting metabolites from osteoarchaeological material. Here, a protocol for extraction and measurement of extracted polar and less-polar/apolar metabolites by ultra-high performance liquid chromatography hyphenated to high resolution mass spectrometry is presented to measure the molecules separated after a reversed phase and hydrophilic interaction liquid chromatography column. Molecular information was obtained, showing that osteoarchaeological material is a viable source of molecular information for metabolomic studies.

## Introduction

Metabolomics is the large-scale study of metabolites and an expanding field that provides an understanding between functional biology (phenotypes) and the inner workings of tissues or whole organisms^1^. In recent years, metabolomics has been employed to evaluate different types of tissues, matrices, and conditions. Metabolites (small molecules with a molecular weight < 1.5 kDa) have been evaluated based on their abundance, transformation, and/or modifications following the impact of different interacting factors in and on organisms, such as age^2^, environment^3^, disease^4^, diet^5^, etc. Metabolomics has been employed on fresh human tissues, which permits researchers to select and determine different groups of interest, and to evaluate the possible effect of covariables that can affect or bias results^6–9^. In contrast to the large number of publications found elsewhere which use metabolomics on fresh human tissue^10–13^, metabolomics in archaeological material has only been used for investigating dental calculus^14^ and ancient artifacts to assess the use of alkaloids by previous populations^15^. To date, metabolomics has not been explored for the analysis of human osteological matrices, even though this approach could reveal novel information about human conditions in the past. In addition, it would provide additional and complementary information to that obtained through the study of different organic and inorganic materials by archaeological scientists. Through this, it is possible obtain direct information about previous populations^16,17^, historical events^18,19^, and past human and animal behaviours^20–23^.

Human osteoarchaeological (skeletal) remains (HSR)—which comprise bones and teeth—are of particular importance in archaeology^24,25^, even if they have gone through various transformations after the death of the organism^24,26–28^, as those elements are the main source of biological information directly related to a past individual. Osteoarchaeologists have used HSR to understand the origins and spread of disease^17,29^, funerary practices^30–32^, human identities^33^, mobility^34,35^, and evolution^36^, among other themes. Osteological literature explaining bone composition, diagenesis, and relevance in the medical and archaeological field is found elsewhere^27,37–40^.

Today, it is possible to derive additional data from HSR material using state of the art analytical platforms, such as genomics^41–45^ and proteomics^46–48^. The different-omics have gained traction through the significant progress of modern analytical tools, such as ultra-high performance liquid chromatography (UHPLC) hyphenated to high resolution mass spectrometry (HRMS)^49^. Additionally, transformational changes to data processing and informatics means that the improved precision and accuracy of the instrumentation has resulted in the detection of a wider repertoire of molecules observed and identified with greater confidence. Moreover, there has been a decrease in the total amount of mass required for analysis, ultimately allowing measurement of samples with low (or ultralow) concentration^50–52^.

The study of HSR from the perspective of small molecules –metabolomics– certainly presents a new opportunity to extract further biological information from past individuals and populations that is informative about phenotype. However, this must be accompanied by the evaluation of the variance related to the abundance of biological metabolites in the HSR, and how this could impact a metabolomic study. Thus, two premises need to be examined. The first relates to changes in metabolites in the human body after death. The second relates to the possible transformation/degradation/disappearance of the biological metabolites in HSR due to exogenous conditions after burial. Following, it is important first to clarify the concept of bone sample.

There are a number of different issues that could prevent the application of metabolomic approaches on HSR. These factors include the different diagenetic and taphonomic processes which transform the bone material after death^27,28^; the interaction of the remains with different air/liquid fluxes in the burial environment^32^; the possible interaction and colonization of microfauna and microflora on the material^53,54^; or the ad/absorption of different molecules in the bone^55^. All these factors can increase the covariance of a metabolomic study and confound interpretation. At the same time, administrative factors, such as obtaining permits from curators to perform destructive micro-sampling on a large set of individuals or obtaining adequate information of excavation and curation processes that could modify/affect the biological information of the individuals in their cleaning, preservation, and storage, could also complicate the convergence of the archaeological and metabolomic fields. Nevertheless, such difficulties have been surpassed in the study of other organic molecules, such as for ancient DNA and proteins^47,56,57^.

To help close the gap between archaeological science studies of HSR and metabolomics, here we propose to undertake the first untargeted study to evaluate the potential of HSR as a source of human biological metabolites. It is important to highlight that this research only considers HSR that have been buried and not impacted by any thermal^58^ or anthropogenic preservation processes^59–61^. To achieve the integration of metabolomics with HSR, we present a detailed micro-sampling method and a robust liquid–solid extraction method to recover aqueous polar and less-polar/apolar metabolites to be utilized in metabolomic studies using UHPLC-HRMS systems. To consider the use of HSR for metabolomic assays as a feasible combination -matrix/technique- this research was divided into four parts. First, we assessed the feasibility of extracting metabolites from HSR and the implications of the complex bone matrix by using macro- and microscopic observations. Second, whether there are differences after chemical liquid–solid extraction in the metabolomic profile of HSR between the two main different bone structures (cortical bone [CB] and trabecular bone [TB]) and the possible variability among different skeletal elements in a single individual. Third, we assessed the impact of the use of post-excavation water cleaning strategies on metabolomic profiles. Fourth, following the considerations found in the three previous parts, we performed an untargeted metabolomic experiment to show the possible outcomes and exemplify the potential of HSR in metabolomic assays for two of the main HRMS platforms currently used: time of flight (TOF) and Orbitrap instruments were employed to measure the metabolomic profile and then evaluate the statistical differences between a set of ancient individuals that show evidence for a particular behaviour (tobacco pipe use) against a control group. With this set of experiments, the analytical procedures, and the overall data presented, it is expected that this research will highlight the potential of HSR matrices in metabolomic investigations to explore past human conditions.

## Experimental section

HSR matrices have not been previously reported for metabolomic studies in the literature and, for that reason, initial experiments to evaluate and obtain a sampling and extraction method to obtain different small molecules on this type of sample were required. Tests with different solvents, solvent volumes, masses of sample, extraction techniques, effect of filtration, among other parameters were evaluated to determine reproducibility and suitability of the methods used. In this manuscript we present—as a guide—one broad and optimal method to sample and extract a large and diverse quantity of small molecules from HSR in a single process for both polar and less polar metabolites for use on LC-HRMS platforms. Nevertheless, it must be said that analytical conditions here described can be adjusted/improved  in future research for specific analytes or analytical platforms.

### Osteoarchaeological samples

Two skeletal collections were used for this research. This included the Medieval Hospital of St John’s the Evangelist in Cambridge. This institute was established at the end of the twelfth century to care for the town’s poor and sick. It closed in the sixteenth century when the buildings passed over to St John’s College, Cambridge. As such, all material predates the use of tobacco and tobacco pipes in England. From this collection, we used disarticulated humeri, femora, and tibiae. The second collection consisted of eighteenth–nineteenth century individuals buried at the former site of Coventry Cathedral (St Mary’s). This cemetery served people from Holy Trinity Church, which would have served the local community. From this collection we sampled femora, ribs, and first and second metatarsals (see Figs. S1, S2, and Table S1). More detail and references on the sites can be found in the Supplementary Information.

### Optical microscopy

A stereomicroscope (Carl Zeiss Microsystems GmbH), equipped with an eyepiece (10 ×), a camera adapter (5 ×), and a set of illumination LEDs (LEICA M205C LEICA Microsystems Switzerland), was used to visualise the different skeletal elements and bone micro-samples. Information was documented by microphotography using Zen Application Suite Version 3.3 (Carl Zeiss Microsystems GmbH). Core samples extracted from femora were detailed using a VARIO microscope (Carl Zeiss Microsystems GmbH) coupled to a motorized 3D stage. Information was captured through Z-stacking by microphotography using the Zen Application Suite Version 3.3 (Carl Zeiss Microsystems GmbH).

### Dry cleaning process for HSR

All bones were subject to a soft, dry cleaning to facilitate visual inspection and guarantee that non-biological material was not introduced during sampling. This was conducted in a clean fume-cabinet by polishing the bone’s surface with a finishing abrasive-buffwheel adapted to a Dremel rotatory tool operated at 5000 RPM. The process was performed until non-biological material was imperceptible. The procedure was carried out without extra pressure on the surface of the bone, avoiding any damage. Detached residues –soil/dust– were collected and properly discarded.

### Micro-sampling of cortical bone structures in HSR

Micro-sampling of cortical bone was performed in a clean fume cabinet as follows. (1) A circular mark to delineate the sampling area of each bone was made using a 6.4 mm diameter glass drilling bit attached to a rotatory tool operated at 5000 RPM. Femora were sampled 10 mm below (inferior to) the midshaft on the anterior surface. This location was chosen to avoid the entheses and measurement points; other bones were sampled at the middle of the total length on the anterior surface. (2) The periosteal bone surface of the marked area was micro-drilled using a high-speed cutter accessory of 4.8 mm diameter attached to a rotatory tool operated at a speed between 5000 to 10,000 RPM. Drilling continued until the inner cortical bone (~ 1–2 mm in depth) was exposed. Removed bone was collected and properly discarded. (3) Exposed cortical bone was drilled, as in step 2, until the mass required for the metabolomics experiments was obtained. Care was taken to avoid sampling the endosteal surface or drilling outside of the marked area. Cortical bone samples were collected and stored in an empty labelled 1.5 mL plastic tube. Biological replicates (triplicates) were extracted for a third of the individuals. Micro-sampling of cortical bone creates a perforation of 6 mm in diameter and variable depth in the skeletal element (see Supplementary Information, Fig. S1). As reference, a single area sampled in a femur will result in approximately 75 mg of cortical bone.

### Micro-sampling of trabecular bone structures in HSR

Micro-sampling of trabecular bone was performed in a clean fume-cabinet as follows. (1) The medullary cavity of the bone was exposed in the region of interest. Tibiae, first and second metatarsals were micro-drilled in the distal areas with a high-speed cutter accessory of 4.8 mm attached to a rotatory tool operated at 5000 RPM, removing the cortical layers, which were collected and properly discarded. Femora were micro-drilled with a glass drilling bit of 6.4 mm in diameter attached to a rotatory tool and operated at 10,000 RPM, removing a complete transversal section of the bone (see Fig. 3) at the distal-posterior surface within the intercondylar fossa, i.e., over the planum popliteum. The trabecular area of ribs was already exposed due to fragmentation. (2) The exposed medullary cavity was carved with a spiral cutting bit attached to the rotatory tool operated at 5000 RPM. Gentle drilling was performed until the total mass required for the metabolomic measurements was achieved, taking care to not affect any other bone structure in the process. Resultant trabecular bone samples were collected and stored in empty labelled 1.5 mL plastic tubes. Biological replicates (triplicates) were obtained for a third of the individuals by increasing the total amount of trabecular bone carved and splitting it later. Micro-sampling of trabecular bone leaves a hole of 6 mm diameter, with the marrow channel exposed (see Supplementary Information, Fig. S1). Total mass obtained by micro-sampling of trabecular bone varies by individual, skeletal element sampled, and carving time. As reference, sampling of an average femur could produce more than 100 mg of trabecular bone, with a carving time of less than 10 s.

### Wet cleaning process for human osteoarchaeological samples

To test the effect of wet cleaning on metabolomics studies, femora, humeri, ribs, and first metatarsals were wet cleaned as follows. (1) Skeletal elements were placed in the laboratory sink and washed for one minute under constant tap water flow at room temperature while being softly brushed. (2) Excess water was removed with a paper towel. (3) Wet bones were air dried in a fume-cabinet at room temperature for one week. The wet cleaning process was performed on a third of the bones originally used in the dry-cleaning process after the initial cortical and trabecular micro-sampling. These holes were covered before wet cleaning using a non-residual tape. Wet cleaned bones were subject to a second process of sampling after drying.

### Filters, vials, and solvents for metabolite extraction and LC–MS measurement

All operations were performed using chemical reagents of optima LC–MS grade. Solvents and formic acid were obtained from Sigma-Aldrich (Poole, Dorset, UK) or Thermo Fisher Scientific (Loughborough, Leicester, UK). Total recovery LC–MS vials were purchased from Waters (Elstree, Hertfordshire, UK). Microcon-10 kDa Centrifugal Filter Unit with Ultracel-10 membrane (Merck Millipore Ltd., Cork, Ireland) and Corning Costar Spin-X centrifuge tube filters with cellulose acetate membranes of 0.22 µm (Corning, Inc, USA) were purchased from Merck.

### Metabolite extraction

**Figure 1:**
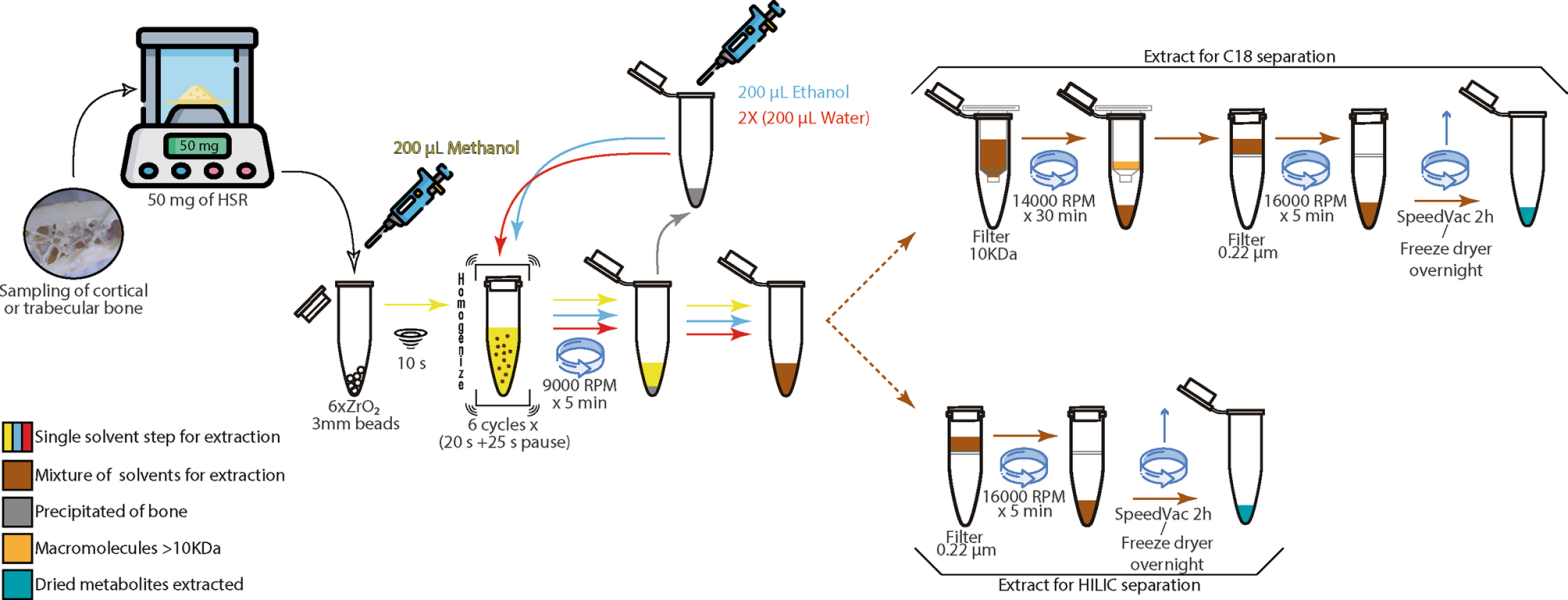
Detailed process for the liquid–solid extraction of polar and less polar/apolar metabolites from human skeletal remains (HSR) for liquid chromatographic separation through C18 and HILIC columns.

Metabolite extraction from cortical and trabecular bone samples were performed in a multistep liquid–solid process as follows (see Fig. 1). (1) 50 mg aliquots of sampled bone were placed in a new 1.5 mL plastic tube with an O-ring cap and 6 ceramic beads (ZrO_2_) of 3 mm diameter. Two-hundred microliters of cold methanol were then added. (2) Tubes were vortexed for 10 s and then placed in a BeadBlaster 24 Microtube homogenizer (Benchmark Scientific Inc., USA) for a sequence of 20 s of homogenization plus 25 s of pause for six cycles. (3) Tubes were centrifuged for 5 min at 9500 RCF. (4) The supernatant was piped out and stored separately. (5) To the initial tube with the precipitate and the ceramic beads, 200 µL of ethanol were added and steps 2–4 repeated. Supernatants collected were mixed. (6) To the initial tube with the precipitate and the ceramic beads, 200 µL of water were added and steps 2–4 repeated. Resultant supernatants were mixed. (7) The alcohol-water solution was split into two halves (Extract A and B). (8) Extract A was filtered through a Microcon-10 kDa centrifugal filter unit—pre-rinsed with water—in a centrifuge for 30 min at 14,000 RCF. (9) To the initial tube with the precipitate and the ceramic beads, 200 µL of water were added and steps 2–3 repeated. The supernatant was decanted and split into two halves; one half was added to the Extract A and step 8 repeated, and the second half added to Extract B. (10) Filtered samples from Extract A and samples from Extract B were centrifuged and filtered through a 0.22 μm membrane (Corning Costar Spin-X). (11) Resultant filtrated extracts were placed in a speed vacuum for 2 h and then frozen with liquid nitrogen to be freeze-dried overnight. Dried extracts were stored at −80 °C. Extraction blank samples were prepared to control the process and guarantee the data quality.

### Sample reconstitution for metabolomic LC-HRMS measurements

To reconstitute the metabolite extracts, a volume of 40 µL of solvent was added to each sample. For less-polar and apolar metabolites analysis by reversed-phase chromatography, a mixture of methanol–water 10:90 was used on the samples labelled as Extract A. For polar metabolites analysis by HILIC chromatography, a mixture of water-acetonitrile 10:90 was used on the samples labelled as Extract B. Liquid solutions were vortexed for 30 s, centrifuged at 20,000 RCF for 15 min at 4 °C, to be transferred in a total recovery glass vial before LC-MS measurement.

### Quality assurance (QA)

To ensure the quality of the metabolomic study, QA procedures were carried out during all workflow steps to reduce the unwanted variation regarding the pre-analytical, analytical, and post-analytical phase of metabolomics^62^. To test and guarantee that the LC–MS instrument was clean and stable for the metabolomic studies, a series of 4–6 injections of pure solvent (injection blank sample) were run at the beginning of each sequence. To evaluate the quality of the extraction process and guarantee that only biological information from the different samples was obtained, extraction blank samples were injected three times randomly during each sequence. To stabilize the MS instrument, pooled quality control (QC) samples, prepared individually for each chromatographic separation technique by mixing 5 µL of each extracted sample after their reconstitution, were injected six times at the beginning of each chromatographic sequence after the injections of instrumental blank samples. In addition, QC samples were injected after each 6–8 randomized samples to monitor the stability of the analytical platform, and after each extraction blank injection to preserve the stability of the MS instrument. To avoid instrumental and statistical bias, all biological samples were randomized before injection. To test the technical variability and possible carry over of the instrument during the injection of the different sequences, a quality control standard mixture (LC–MS QC STD Part number 186006963-1, Waters) was injected at the beginning and at the end of each sequence and t_R_ and ions evaluated.

### Untargeted metabolic fingerprinting by high-flow-UPLC‑IM-TOF‑HRMS

Untargeted analyses were performed on a high flow Acquity UPLC system coupled to a Waters Synapt G2 HDMS system (Waters Corporation, Manchester) composed of an electrospray ionization (ESI) source (operated either in positive mode or negative mode), an ion mobility (IM) cell, and a time-of-flight detector (TOF). MS data was collected using the mobility MSe function in profile mode (full scan) over the m/z 50–1500 range. Capillary voltage was set at 3.00 kV, sampling cone 30 V, source temperature 120 °C, desolvation temperature 600 °C, and desolvation gas 1000 L/h. IM and TOF cells were calibrated in advance and lock spray of leucine enkephalin was used and infused at 10 µL/min. Chromatographic separations were carried out by using both a reversed phase (RP) C18 column (Waters Acquity UPLC BEH C18 column (2.1 × 100 mm, 1.7 µm)) for semi polar and apolar metabolites, and a hydrophilic interaction liquid chromatography column (Acquity UPLC BEH HILIC (2.1 × 100 mm, 1.7 µm)) for polar metabolites. Samples were maintained at 4 °C in the auto sampler and aliquots of 5 µL injected per run. The flow rate was set at 0.4 mL/min, and the column temperature was maintained at 40 ± 2 °C. Solvents used for separation consisted of 0.1% formic acid in water as mobile phase A, and 0.1% formic acid in acetonitrile as mobile phase B. The RP gradient elution program started running at 2% B, held for one minute, then mobile phase B was linearly increased to 20% to minute 5, 25% in minute 6, 75% in minute 7, and 98% in minute 7.5, then held at 98% of B until minute 8, going back to initial conditions of 98% of A in minute 9, and equilibrated the column until minute 10. The HILIC gradient elution program started running at 3% A and held for one minute, then mobile phase A was linearly increased to 20% in 3 min, 25% in one minute, 75% in two minutes, and 97% in one minute, then was held for half a minute, going back to initial conditions of 97% of B in 1.5 min to later be equilibrated for four minutes.

### Untargeted metabolic fingerprinting by nano-UHPLC‑Orbitrap‑HRMS/MS

These analyses were performed using a Dionex Ultimate 3000 NCS-3500RS Nano Proflow UHPLC system hyphenated with a high-resolution hybrid quadrupole Orbitrap tandem mass spectrometer (Q Exactive HF, Thermo Scientific). Sample extracts were stored in the autosampler at 8 °C and 1-µL loaded at 300 nL/min on a nanoflow UHPLC column (PepMap RSLC, C18, 2 µm, 100 Å, 50 µm × 150 mm, Thermo Scientific). Chromatographic separation of metabolites was achieved at 40 °C and by applying a biphasic gradient consisting of mobile phase A (2% acetonitrile) and phase B (98% acetonitrile), both containing 0.1% formic acid. The RP gradient elution started at 10% B (0–2 min) and a shallow gradient to 95% B over the following 15 min. The high organic (95% B) was kept for 10 min with a 0.5 min return to starting conditions (10% B), followed by 12.5 min of equilibration time, leading to a total runtime of 40 min. Eluted metabolites were ionised by positive electrospray ionization (1.70 kV) using an EASY-Spray ion source (Thermo Scientific). The capillary temperature was kept at 250 °C and an S-lens RF level was 50 V. Orbitrap acquisition was done in either full scan mode for quantification or iterative data-dependent fragmentation (ddMS2) mode for identification^63^. Full scan acquisition was recorded using a resolution of 240 k at m/z 200, an automatic gain control (AGC) target of 1e6, a maximum injection time of 100 ms, and a scan range of m/z 120–1500. ddMS2 acquisition was carried out using the same full scan settings and MS2 resolution of 15 k, maximum IT of 50 ms, isolation window of 1.0 m/z, AGC target of 1e5, loop count of 10, and stepped collision energies of 15 and 70 NCE. The acquisition was performed with a dynamic exclusion of 20 s, minimum AGC target of 1000, charge exclusion of > 2, and an apex trigger between 6 and 12 s. Sub-ppm mass accuracy was ensured by real time calibration from lock mass of 371.10124 (polysiloxane from air).

### Data treatment

An initial pre-processing treatment—run alignment, ion detection, peak picking, isotope and adduct deconvolution, ion drift measurement, etc.—was performed on the raw data from the different LC-HRMS platforms to obtain the compound metabolomic matrices for the different experiments in all the platforms. Pre-processing treatment was performed using Progenesis-QI (Nonlinear dynamics) software for the resultant data from the HF-UPLC‑IM-TOF‑HRMS instrument. Compound discoverer 3.3 (Thermo Scientific) software was used for the resultant data from the nano flow-UHPLC-Orbitrap-HRMS platform, following the “untargeted metabolomics with statistics and detect unknowns with ID using online databases and mzlogic” workflow. Workflows for both instruments were processed, including on each experiment, the biological samples and quality assurance samples (i.e., pooled QC, extraction blanks, and injection blanks). Resultant data matrices containing the different samples and the total list of compounds detected for each experiment were inspected manually by using Microsoft Excel 2016 to filter out non-biological information (noise, instrumental signals, compounds from the extraction process, etc.). In short, the average value was calculated for each compound in the data matrix for the pooled QC, instrumental blank, and extraction blank samples, as well as the relative standard deviation (RSD) for the QC samples. Compounds with an injection or extraction blank contribution greater than 5% with respect to the average QC sample were removed. Compounds with a QC RSD value greater than 40% were removed. Before any further statistical analysis, data transformation was applied to the different data matrices; K-nearest neighbours based on similar features was employed to replace missing values; and mean sample normalization; log10 data transformation; and pareto data scaling applied to the different sets of data.

### Statistical analysis

To establish the overall differences in the metabolic profiles between groups studied, multivariate statistical analysis, including unsupervised principal component analysis (PCA) and supervised partial least squares discriminant analysis (PLS-DA), were performed. MetaboAnalyst 5.0 (https://www.metaboanalyst.ca/) was employed for the statistical analysis of all the experiments, as well as SIMCA version 14.0 (Umetrics) software for confirming the results for the untargeted metabolomic analysis comparing a healthy versus a condition group. The quality of the established statistical models was evaluated using the R^2^X, R^2^Y, and Q^2^ parameters. A permutation test was performed in 100 cycles to evaluate the possible overfitting in the PLS-DA models. PCA models were performed, including QC samples, to evaluate stability of the analytical system and the data quality, using as an indicator the clustering of QC samples in successive injections during the sequences. To visualize significant features up-regulated or down-regulated when two selected groups of interest were studied, univariate statistical tests were performed, and volcano plots were drawn by transforming the fold change (FC) value of each substance peak to log2 (FC) and transforming the P value (P = 0.05) of Student’s t-test to − log_10_(P value).

### Mummichog pathway analysis

The full list of features found by each analytical platform without prior peak annotation were mapped to pathways using the mummichog algorithm in the opensource web-based tool MetaboAnalyst 5.0 (https://www.metaboanalyst.ca/). Tests to evaluate the accurate masses of features in the affected pathways in databases from a pair-wise comparison between the control and treatment group were performed by using, individually, C18 or HILIC data matrices, as well as combining both sets of data in a single matrix to compare with the reviewed known pathways presented in the human repository from the KEGG^64^ and BioCyc libraries. The mass difference between imputed metabolites and the database metabolites was set at 1 ppm and the significant threshold for metabolites selection was 0.25.

## Results and discussion

The average adult human skeleton is composed of 206 bones^37^ and, during life, each bone is under constant regeneration as a dynamic tissue^37–39^. Bones are made up of a mixture of organic components (~ 30%; i.e., osteoblasts, osteoclasts, osteocytes, and osteoprogenitor cells) in a hard and resistant inorganic inter-cellular matrix (~ 70%, up to 25% water, and 50% mineral salts, mainly bio-calcium-hydroxyapatite and bio-calcium-phosphate)^37^. Human bone tissue is structured by a different arrangement of osteological aggregations, with the simplest classification split into two different structures: cortical bone (CB) and trabecular bone (TB)^37^. Cortical bone has a periosteal bone layer (PB) which is the most external cortical structure. PB is bonded and in direct contact with muscles, tendons, and ligaments, varying its extension with the total surface of each skeletal element. CB is a compact structure that gives bone rigidity and allows the flux and exchange of different biological material along and from/to the inner part of the bone through capillaries, blood vessels, and nerve fibres. CB relative abundance varies depending on the skeletal element, being the main component in long bones. TB is a honeycomb-like irregular structure located within CB. TB is a protected structure that confines different blood vessels and red and yellow marrow^7^, and is present in all bones, being the dominant structure in irregular and flat bones^37^.

Post-mortem, the skeleton undergoes changes that occur within different time frames^28,31,47,65,66^, leading to a variation in the quantity and concentration of organic matter in the bone with respect to the living individual^65^. These changes are: (1) osteonecrosis, which is the process that triggers the necrosis of the different cells in the bone tissue by changes in the extracellular or intracellular microenvironment, which is promoted by the cessation of the flux of veins, arteries, and capillaries, together with the impossibility of eliminating the irreversibly damaged cells^65,67^; (2) a degradation process of the resultant molecules after necrosis, this being dictated by the burial environment—pH, humidity, temperature, oxygen, and water availability, the presence or not of microorganisms, and the transformation of the different phases of the bone matrix—organic and/or inorganic^27,47,54^; and (3) due to the high thermodynamic disequilibrium between the bone and the burial environment, an exchange of components can take place over time^55,56^. These changes, as of yet, have no direct measure that allow us to relate the concentration in the tissue with the post-mortem interval. From a metabolomic perspective, all could result in a decreased concentration and/or a change in the type of metabolites present (or molecular fingerprint) in bone tissue after death in comparison to the peri-mortem condition. Yet, the bone matrix could also preserve small molecules which may be by their size, shape, or affinity retained by the different components of the bone matrix, i.e., collagen, proteins, salts, etc. Small molecules resultant of osteo-cells trapped in the lamellar areas during bone remodelling, or metabolites deposited/adsorbed on the surface of the different porous channels and trabeculated areas of the bone after the osteonecrosis processes, are expected.

In the next sections, we assess the viability of HSR as a feasible matrix for metabolomic assays and the different considerations to be made in an experimental design before using this type of material.

### Covariance and experimental design considerations before sampling and extraction of metabolites in HSR

To study the metabolomic variability in bone structures and skeletal elements, two archaeological collections were used (see Materials and methods and Figs. S1, S2). A total of 57 bones (comprising humeri, femora, tibiae, metatarsals, and ribs) were assessed, which first were subject to osteological inspection, dry cleaning, and optical microscopy.

**Figure 2:**
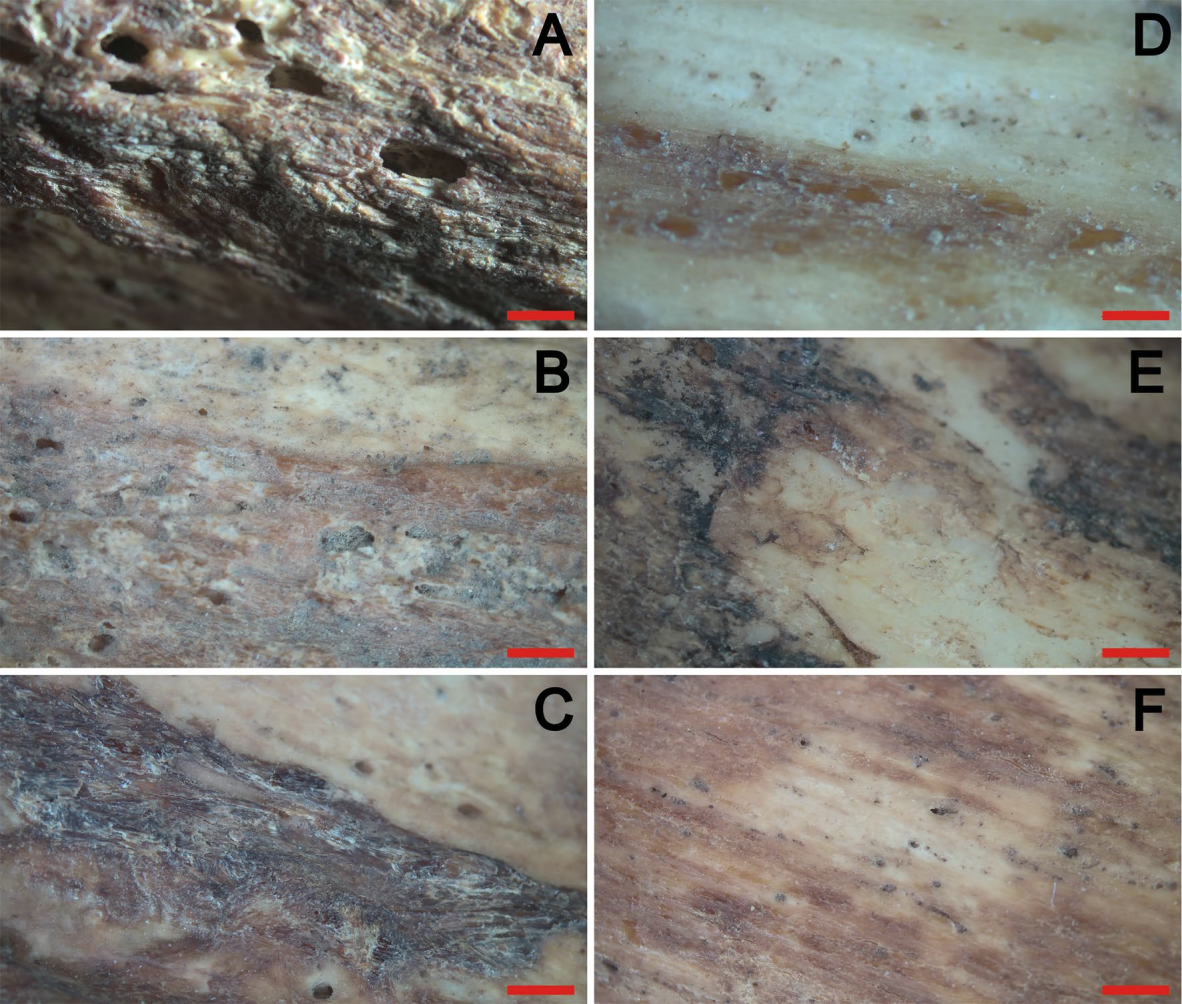
Periosteal bone of human archaeological femora. Microphotographs of periosteal bone of two human femora in different locations, as an example of the heterogeneity of the periosteal bone in terms of staining and presence of remaining soft tissue attached to the bone. (**A**–**C**) Individual SK400; (**D**–**F**) Individual SK4. Red lines indicate a scale of 2 mm in length.

The different HSR material present characteristics of well-preserved archaeological dry material (no cortical cracking or flaking), being completely different to fresh tissue (green bone) regarding texture, density, humidity, etc. PB morphology, texture, colour, and composition in the HSR varied randomly among samples, even for the same skeletal elements. PB presented different types of staining and non-osseous solids attached to the surface without regular pattern. Staining was associated with diagenetic and bioerosion processes; meanwhile solids were associated with remaining soft tissue (i.e., muscle, ligaments, etc.) from incomplete degradation processes. Figure 2 shows two femora which show the heterogenicity of PB surfaces. These observations suggest that PB is a complex matrix that could offer various qualitative information related to the skeletal element and any residual soft tissue attached to it. As a result, the quality of this bone structure for metabolomic assays is low as it is impossible to quantify or classify such variation.

To find HSR samples with a more controlled and homogenous composition, CB and TB structures were evaluated. To do this, fragmented bones –i.e., bones with exposed internal structures through post-mortem fractures, apertures by acidification, flaking, etc.—were examined. The visual and microscopic osteological evaluation showed CB and TB structures are affected by direct contact with the burial environment. Fragmented CB structures presented staining on the post-mortem exposed surfaces, which varied in colour and depth from sample to sample. Fragmented TB presented black staining and the presence of soil, vegetal, and fungal material trapped in their pores. Alterations to CB and TB in fragmented material were present in all skeletal elements randomly, which limited their potential for metabolomics due to the presence of foreign contamination. Nevertheless, CB and TB structures in HSR material that presented fractures occurring during excavation, post-excavation, and storage processes presented an absence of staining associated with the burial, nor the presence of any remaining non-osseous material, or alteration in their structures. Such observations indicate that both CB and TB could be used for metabolomic studies if from bones that are not broken prior to excavation.

**Figure 3:**
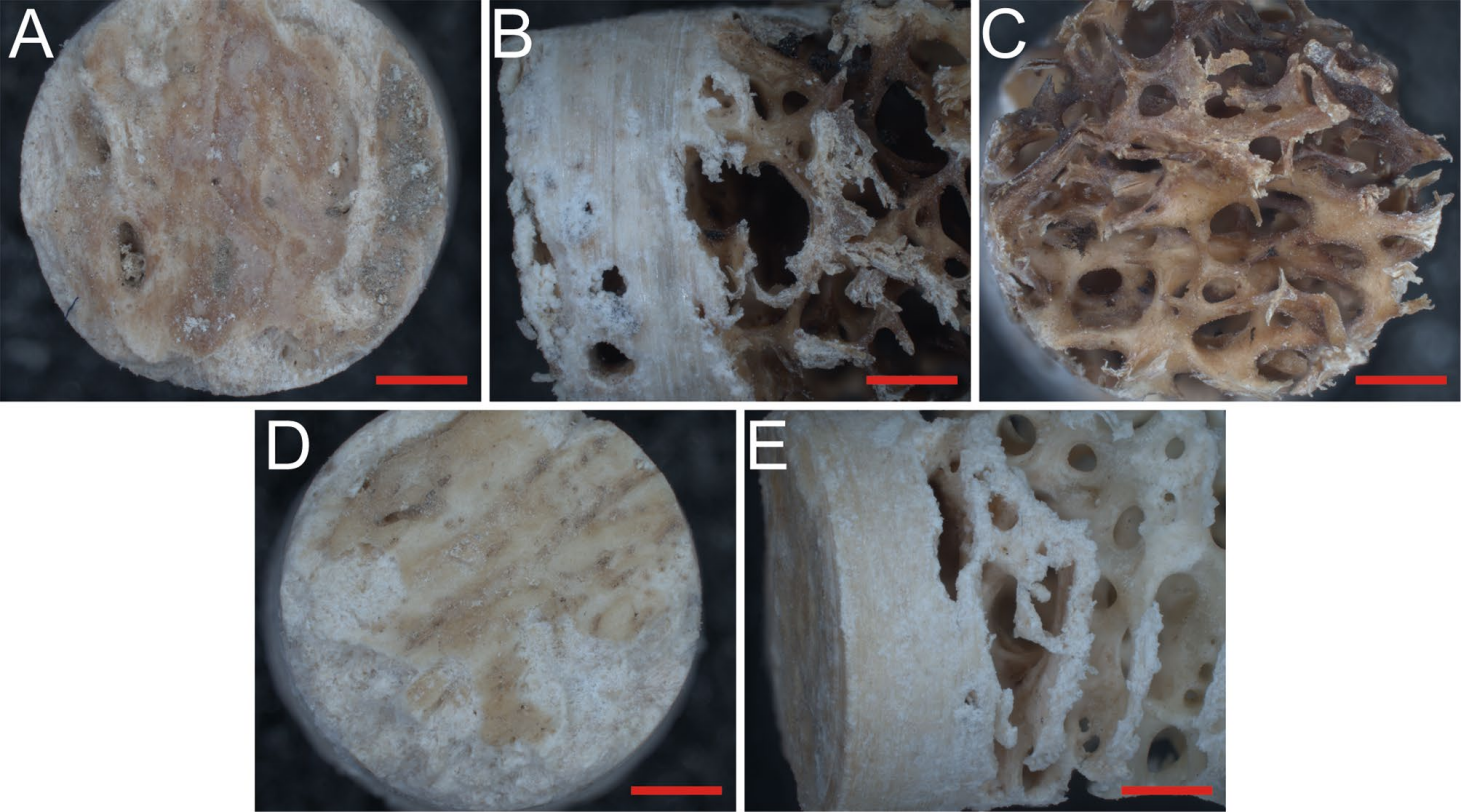
Human osteoarchaeological femora core micro-samples. Microphotographs of unfragmented femur bone cores extracted from two individuals showing the sequence of bone structures in this skeletal element, its distribution, and variation in appearance in human osteoarchaeological material. (**A**–**C**) Individual SK1089; (**D**,**E**) Individual SK2. (**A**,**D**) Detail of periosteal bone from a superficial view; (**B**,**E**) lateral view showing, from left to right, the periosteal, cortical, and trabecular bone structures; (**C**) detail of trabecular bone from a deep view. Red lines indicate a scale of 1 mm in length.

To evaluate the quality of CB and TB structures, unfragmented HSR –i.e., bones without fractures– were subject to a micro-stratigraphic cut to obtain cylindrical cores that contained both structures. Figure 3 shows an example of resultant cores from two femora. In these, the sequence of bone structures was evident: an initial PB layer, followed by CB, and finally the TB structure. The cores allowed us to determine that diagenesis mainly affected PB, as CB and TB appeared less altered after the individual’s death. Light microscopy and scanning electron microscopy, coupled with electron dispersive spectroscopy measurements on HSR core micro-samples, showed that CB is a compact structure of whitish colour, with micro-channels and a chemical composition based on phosphorus and calcium elements, proper of a hydroxyapatite material (see Supplementary Information Fig. S3A). In contrast, TB presented an irregular macroporous structure of variable colour, from whitish to an intense red colour, with a chemical composition of iron, magnesium, calcium, and phosphorous (see Supplementary Information Fig. S3B), indicating that this structure corresponds to a bone tissue, with the presence of degradation products related to different blood/marrow cells^39,68^. These observations coincide with reports of fresh bone, relating to porosity in bone structures^69,70^. Considering that CB and TB are subject to bone remodelling processes in which different osteo-cells could be trapped inside the bone lamellar layers^39^, it is possible to postulate that some metabolites from different osteo-cells, along with metabolites from blood/marrow degradation, could remain through chemical or physical forces in the HSR even if at low concentrations. This makes this type of material a potential source for metabolomic analyses. However, as each bone structure has different chemical environments and variable total surface area related to its role in physiology, it is reasonable to consider that differences in metabolite composition/concentration between structures might be expected. This implies that the metabolomic profile of each structure could differ, presenting different biological information of the condition of the individual during life.

An untargeted metabolomics approach using CB or TB separately –instead of mixed as a single sample– is an optimal part of our experimental design to allow for the evaluation of the different metabolites present or not in the two major structures of bone. This should reduce the variance among observations from individuals for which there are already present a series of unknown confounders (e.g., body mass index, exact age, diseases, etc.). This will help in the application of statistical models to find and explain the differences among studied groups. Nonetheless, for future investigations, researchers should evaluate their own requirements, noticing that the use of PB structures, or samples that mix the different structures, will likely increase the variability of the experiment, and will limit the quantitative data generated, even if their use could provide extra qualitative data. To validate our previous argument, both a micro-sampling and a liquid/solid extraction method were developed to recover the aqueous polar and less-polar/apolar metabolites in HSR for metabolomic analyses.

### Cortical and trabecular micro-samples of HSR for untargeted metabolomic studies

Microscopic observation of CB and TB micro-samples from different skeletal elements in all individuals allowed us to undertake an initial investigation of this material before its chemical analysis. The different skeletal elements presented similar microscopic characteristics for each bone structure. Figure S4A,B shows typical examples of the material obtained for CB, which consists of a white-cream solid compact grain material of a homogeneous size. Figure S4C,D shows an average example for the TB sampling, consisting of a flaked porous material of irregular size with pale to red-ochre coloration. This demonstrates that, microscopically, the matrix for each bone structure is not equal, potentially inferring that the molecular content could also be different, even if after the liquid/solid extraction both structures produce transparent solutions.

### Variance of metabolomic profile in HSR material by skeletal element and structure in an untargeted metabolomic study

In archaeology, it is not always possible to have access to the same skeletal element for destructive sampling. Here, we explore for differences that could affect an untargeted metabolomic study of HSR when different skeletal structures and/or elements are used.

**Figure 4:**
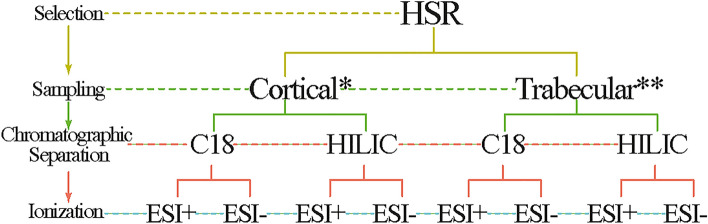
Scheme of the eight experiments performed in human skeletal remains (HRS) to evaluate the differences in skeletal structures by untargeted metabolomic LC-HRMS. *Cortical bone from femur, humerus, tibia. ** Trabecular bone from 1st metatarsals, 2nd metatarsals, rib.

To evaluate whether there was any variance in the HSR metabolomic profile using different skeletal elements in an untargeted LC-HRMS metabolomic study, eight experiments were performed (see Fig. 4) on six different skeletal elements, three for cortical bone and three for trabecular bone (see Materials and Methods). The selection of the skeletal element for each evaluated group was based on the CB/TB ratio for each bone, and the regular abundance of this material in archaeological findings. The first group was composed of CB from femora, humeri, and tibiae, and the second group was composed of TB from ribs and first and second metatarsals.

Analysis of the metabolomic profiles after data processing and data cleaning of the MS matrices proved that, as proposed, HSR material contains various metabolites that can be recovered because of the sensitivity of the LC-HRMS platform used. A comparison of the results from the two different separation columns showed that a higher number of polar compounds were detected than less-polar/apolar compounds in both HSR structures, but the total number of compounds found for CB was higher than TB after all the measurements. This confirms that bone structure is a factor of variance, and that the use of separate structures in metabolomic studies in HSR is an optimal approach. Additionally, results demonstrated that modification to the instrumentation could result in detecting a different number of compounds, and improvements are possible, as was the case for the ionization mode, which after using the ESI in positive mode offered a higher number of compounds than in negative mode.

To obtain a statistical model which allowed us to easily interpret the metabolomic profiles of the different samples, and to find a pattern or phenotypic differences between groups without prior knowledge, unsupervised principal component analysis (PCA)^71,72^ was employed on the post-processed and cleaned data matrix for each experiment (see Materials and Methods). Furthermore, partial least-squares discriminant analysis (PLS-DA)^73^ was used to evaluate the different sets of metabolomic results. However, even though PLS-DA models presented an optimal separation among groups, these results were discarded as its cross-validation and permutation tests indicated a high rate of false-positives^74^, as well as overfitted models. This was probably due to the use of a small number of observations, the large set of variables, and the low variance among groups, which could bias the statistics^75,76^. For the above, only PCA models are considered and evaluated next.

**Figure 5:**
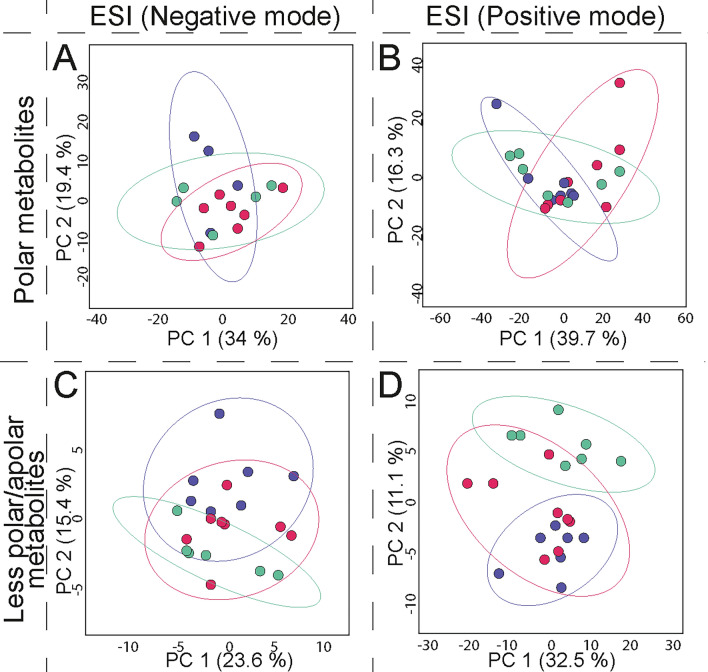
Principal component analysis for untargeted metabolomic tests using cortical bone (CB) from different human skeletal elements. Score plots for metabolites extracted and measured through HF-UPLC-IM-TOF-HRMS from cortical bone of femora, humeri, and tibiae of disarticulated human osteoarchaeological remains. Data matrices were subject to data cleaning, normalized, transformed, and scaled (see Materials and Methods section). (**A**) Polar metabolites (HILIC, ESI negative mode), N = 27, 471 variables; (**B**) Polar metabolites (HILIC, ESI positive mode), N = 28, 1629 variables; (**C**) Less-polar/apolar metabolites (C18, ESI negative mode), N = 27, 154 variables; (**D**) Less- polar/apolar metabolites (C18, ESI positive mode), N = 28, 665 variables. Ellipses indicate 95% of confidence for each group. The explained variances of selected principal components are shown in brackets. Observations are coloured according to each skeletal element: femur, red; humerus, green; tibia, purple.

**Figure 6:**
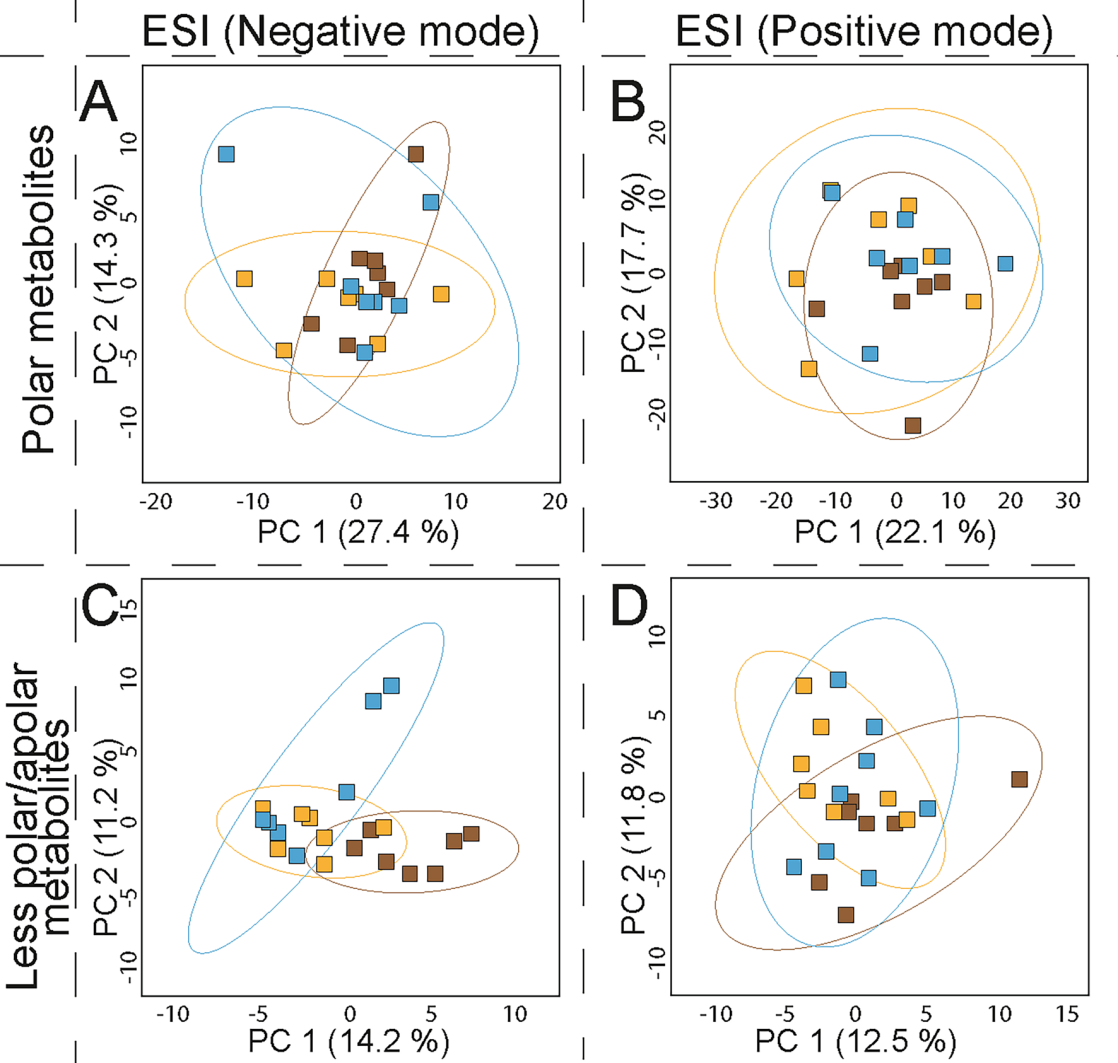
Principal component analysis for untargeted metabolomic tests using trabecular bone (TB) from different human skeletal elements. Score plots for metabolites extracted and measured through HF-UPLC-IM-TOF-HRMS from trabecular bone from first and second metatarsals, and ribs of disarticulated human osteoarchaeological remains. Data matrices were subject to data cleaning, normalized, transformed, and scaled (see Materials and Methods section). (**A**) Polar metabolites (HILIC, ESI negative mode), N = 27, 471 variables; (**B**) Polar metabolites (HILIC, ESI positive mode), N = 27, 1395 variables; (**C**) Less-polar/apolar metabolites (C18, ESI negative mode), N = 27, 243 variables; (**D**) Less-polar/apolar metabolites (C18, ESI positive mode), N = 34, 312 variables. Ellipses indicate 95% of confidence for each group. The explained variances of selected principal components are shown in brackets. Observations are coloured according to each skeletal element: 1st metatarsals, yellow; 2nd metatarsals, blue; rib, brown.

The PCA models obtained for the different untargeted metabolomic profiles of the skeletal elements under the different analytical platforms using CB and TB extracts are shown in Figs. 5 and 6, respectively (see also Fig. S5). The principal component analysis presented a low accumulated variance score in all tests, being lower in TB models than for CB. This result suggests a degree of similarity among metabolomic profiles of skeletal elements. PCA models also showed metabolomic profile variance and different trends by skeletal element. This variance increased when the ESI was used in positive polarity, which detected a higher total number of molecules. Dispersion among observations in the different groups for both CB and TB models reflects a native variance of the samples from the same skeletal element. PCA models for the different metabolomic profiles allows us to propose that variance among observations can be related to the individual’s biology, rather than to diagenetic or burial influences, for example. No evidence for clustering or patterning of data that corresponded to the fact that all individuals shared the same type of burial conditions and similar diagenesis time were found.

These results indicate that, even if the metabolomic composition of different skeletal elements is similar, they are not exactly the same. They show a variance which could be interesting to study further when confounders are controlled for in a metabolomic experiment. This simple observation has a deeper repercussion in the consideration of the use of HSR in archaeological studies, as this implies the need for the researcher to think about the availability of individuals and skeletal elements, which could reduce the total number of individuals available for such studies. Using a mixed sample of bone types could potentially have a significant impact on the reliability of any results drawn.

In analysing both groups of experiments for CB and TB, it was evident that metabolic profiles for polar compounds presented lower dispersion between skeletal structures than the less-polar/apolar compounds. This result can be explained if we consider the constant interaction between the HSR and the burial environment. Polar metabolites in the bone could be easily impacted by external factors, such as the constant flux of liquids in the ground –from the individual’s initial burial until their bones are excavated–, leading to a constant exchange of compounds until an equilibrium is reached. On the contrary, less-polar/apolar compounds, which are more likely to be retained in the bone due to their lower affinity with the highly polar aqueous media present in normal burial conditions, are more likely to be retained in the bone matrix but could be changed by concentration gradients.

Metabolomic studies using fresh tissue from living individuals, such as blood, marrow, and osteological tissue^7,10,77,78^, have considered biological confounders (e.g., age, biological sex, body weight, ethnicity, smoking status, diseases, etc.) and have taken steps to reduce variance through careful individual selection in each analytical trial. In the case of HSR, such a precise selection can rarely be made, and a relatively high natural variance among observations must be accepted, whereby different diagenetic processes can produce changes in the metabolites present in the tissue. Therefore, we consider that an optimal strategy to reduce the variance in the study of HSR is to perform a metabolomic study where micro-sampling, bone structure, skeletal element, and spatial location of the bone remains consistent across different individuals, allowing the achievement of more reliable information that can be compared statistically to establish differences among the evaluated populations.

### Variance of metabolomic profiles in water-cleaned HSR material by skeletal element and structures in an untargeted metabolomic study

Following recovery, archaeological HSR can be subjected to multiple post-excavation processes. Some of these operations use different chemical reagents to clean, consolidate, or stabilize the bone material, with the use of water to clean the most common practice^79^. As metabolomic extracts from HSR could provide a close approximation to the final stage of life of the individual, use of further chemical reagents on bone can disturb the metabolic information, and data drawn from resultant metabolites could be unreliable. By using the same individuals as in the previous experiment, the repercussions of the use of (tap)water to clean bones on the metabolomic profile was evaluated. Three experiments were performed: the first assessing CB from humeri and femora; the second sampling TB from first metatarsals and ribs; and the third sampling TB from femora. The latter was selected as the TB is highly protected by the thick femoral CB in comparison to other bones. The LC-HRMS experiments were followed, as before, by using only ESI in positive mode to increase the sensitivity of the method and expand the variance of the measurement.

After the HF-UPLC-IM-TOF-HRMS measurement, the metabolomic data matrices were subject to data cleaning and later used to obtain the respective PCA models. Statistical models were built to better evidence the variance in the metabolomic profiles of the different samples before and after the use of water as a post-excavation cleaning process on CB and TB. Resultant PCA models are presented in Fig. S6.

The PCA models show that post-excavation water treatment has ramifications for the metabolomic profile of different skeletal elements for both CB and TB. Samples not subject to water treatment conserved a variance between skeletal elements, providing corroboration that an influence in the variance of the metabolite profile for each skeletal element does exist. The two skeletal elements studied for CB after wet cleaning treatment (i.e., femur and humerus) presented a close clustering among observations in the less-polar/apolar metabolomic profile, with a shift towards positive values of the PC1 on the PCA model with respect to the untreated samples. In the polar compounds, CB samples for both skeletal elements also presented a decrease in variance among observations, but they did not shift from the untreated samples. This suggests that both skeletal elements are affected by the wet cleaning, resulting in the material presenting with a similar metabolomic composition between individuals. The two skeletal elements studied for TB after wet cleaning treatment (ribs and first metatarsals) presented a decrease in variance among the treated observations and a positive shift towards the PC1 component with respect to the untreated samples for the less-polar/apolar metabolites, and a negative shift towards the PC2 for the polar compounds with respect to the untreated samples. Nevertheless, the skeletal elements still split from one another, with the rib observations presenting with the highest degree of change. This result suggests that wet cleaning affects the TB metabolomic profile, and the degree of change can vary from element to element, likely due to the relative differences in the thickness of CB, which can protect the TB from water to some degree. Finally, wet cleaned TB from femora showed a change in their metabolomic profile with respect to the untreated samples; they presented an increase in variance among observations. When comparing the PCA model for the TB samples from the femur against that from the ribs and metatarsals, it was evident that the femur presented a completely different behaviour after the wet cleaning, potentially indicating that the thickness of the cortical bone structure plays an important role in protecting TB metabolites.

The previous results for CB and TB observed in the different PCA models for the wet cleaning (i.e., changes of variance among the different observations and differences in the metabolomic profiles) suggest that this operation could potentially alter the HSR material, producing a material with homogenous metabolomic profiles among individuals. If this type of treated material is used in a metabolomic study, this could have ramifications for data interpretation, as the samples will have an altered metabolomic profile and the biological information of the individuals will be modified, making it difficult to apply a correct statistical model.

### Untargeted metabolomic analysis of HSR, comparing a control versus a condition group by the use of two different HRMS platforms

The results presented show that HSR are suitable for retrieving metabolomic profiles of peri-mortem conditions in past populations by LC-HRMS measurements, and that our micro-sampling and metabolite extraction methods are suitable for this type of research. In order to establish whether we could use the developed untargeted metabolomic approach in archaeological HSR, we collected femoral CB and TB extracts from 5 individuals presumed to have not been exposed to tobacco (control) and 5 individuals who consumed tobacco (test). Each group (n = 5) were controlled for age, ethnicity, and post-mortem processes by choosing only males with a similar age (35–50 years) from Britain with no evidence of post-excavation manipulation. The test group were individuals with evidence of a dental modification associated with tobacco pipe use from Coventry, UK; meanwhile, the control group consisted of individuals that pre-dated tobacco from Cambridge, UK. This pilot study was performed using the same instrumentation in the same facility as in the previous sections at the van Geest MultiOmics facility of the University of Leicester (UK). Measurements were conducted in a HF-UPLC-IM-TOF-HRMS using both C18 and HILIC columns, and compared with measurements of the same extracts in a second facility located at the Environmental Metabolomics Lab, Dept. of Environmental Science of the Aarhus University (Denmark). Here, measurements were conducted through a sensitive nano-UHPLC-Orbitrap-HRMS/MS with a nanoflow (300 nL/min) C18 column. This allowed us to validate our extraction method and permitted us to evaluate the results by the two main HRMS instruments used in metabolomic research. Both facilities followed the LC-HRMS measurements using positive ionization mode (ESI +) to explore the metabolomic profile of a health parameter of previous populations. The use of the Q-TOF (Waters) and Orbitrap (Thermo Scientific) systems presented here for the analytical measurement together with their respective commercial software (Progenesis QI and Compound Discoverer) for the post-analytical processing, demonstrate the potential of two of the most common LC-HRMS platforms in metabolomic research when dealing with complex matrices such as HSR material. Previous work by others have evaluated differences between HR MS platforms and concluded that differences do emerge between these platforms^80,^ which also demonstrate a degree of complementarity. Our data corroborates this previous literature and indicates that whilst a large degree of molecular alignment occurs between the platforms, there are differences which need to be carefully assessed.

After extraction of polar and less-polar/apolar metabolites, LC-HRMS measurement, and data cleaning, 444 (C18 TOF), 1283 (HILIC TOF), and 1780 (C18 Orbitrap) compounds were obtained for the CB samples, and 192 (C18 TOF), 689 (HILIC TOF), and 1623 (C18 Orbitrap) for the TB samples. The difference between the Orbitrap-MS and the TOF–MS system can be attributed to the different resolutions and sensitivities in the MS instruments, the selected experiment and the difference in the internal diameter of the columns, where the nano-LC system enhances the sensitivity of the measurement^81^. This increases the possibility to detect compounds that could be suppressed by the signal/noise ratio of the Orbitrap instrument or hidden by the influence of other factors, such as the solvent blank, or the blank of extraction compounds.

To evaluate the different metabolic profiles obtained through the two platforms, PCAs from each analytical method were evaluated with the inclusion of pooled quality control (QC) samples and the QC samples for each group (i.e., one for the control samples and another for the affected group) (see Supplementary Information, Fig. S7). Pooled QC samples were grouped in a single cluster for each PCA model, indicating that the quality of acquired data was optimal and separation in the different samples can be related to biological –and not analytical– variation. QC samples containing aliquots from only one group did not cluster together with the QC pooled samples. Instead, each sample displayed a displacement on the PCA score plots, with similar tendency to the observations of each respective group, indicating that, effectively, each group contains a characteristic metabolomic profile (see Supplementary Information, Fig. S7).

**Figure 7:**
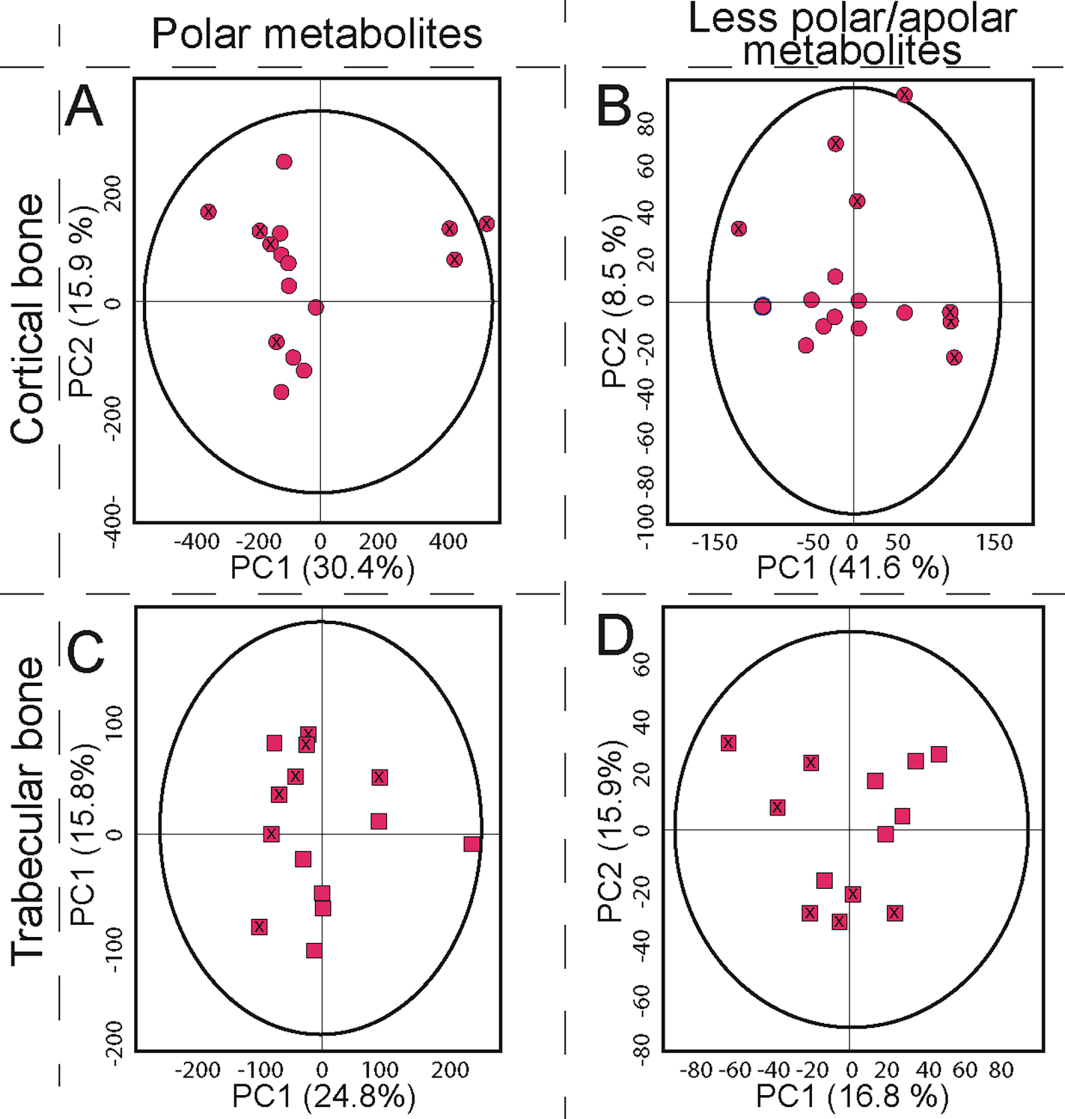
Principal component analysis of the untargeted metabolomic tests of femora, comparing an affected group with a control group using cortical and trabecular samples from human osteoarchaeological remains in a UPLC-IM-TOF-HRMS ESI positive mode platform. (**A**) Polar metabolites in cortical bone (HILIC) R^2^(cum):0.836, Q^2^(cum):0.621, N = 17, 1629 variables; (**B**) Less-polar/apolar metabolites in cortical bone (C18) R^2^(cum):0.655, Q^2^(cum):0.442, N = 17, 665 variables; (**C**) Polar metabolites in trabecular bone (HILIC) R^2^(cum): 0.707, Q^2^(cum): 0.393, N = 14, 1395 variables; (**D**) Less-polar/apolar metabolites in trabecular bone (C18) R^2^(cum):0.472, Q^2^(cum):0.0944 N = 14, 312 variables. X-Dots represent affected individuals in cortical bone. Dots represent individuals from the control group in cortical bone. X-squares represent affected individuals in trabecular bone. Squares represent individuals from the control group in trabecular bone. Ellipses indicate 95% of confidence for the PCA model. The explained variances of selected principal components are shown in brackets.

**Figure 8:**
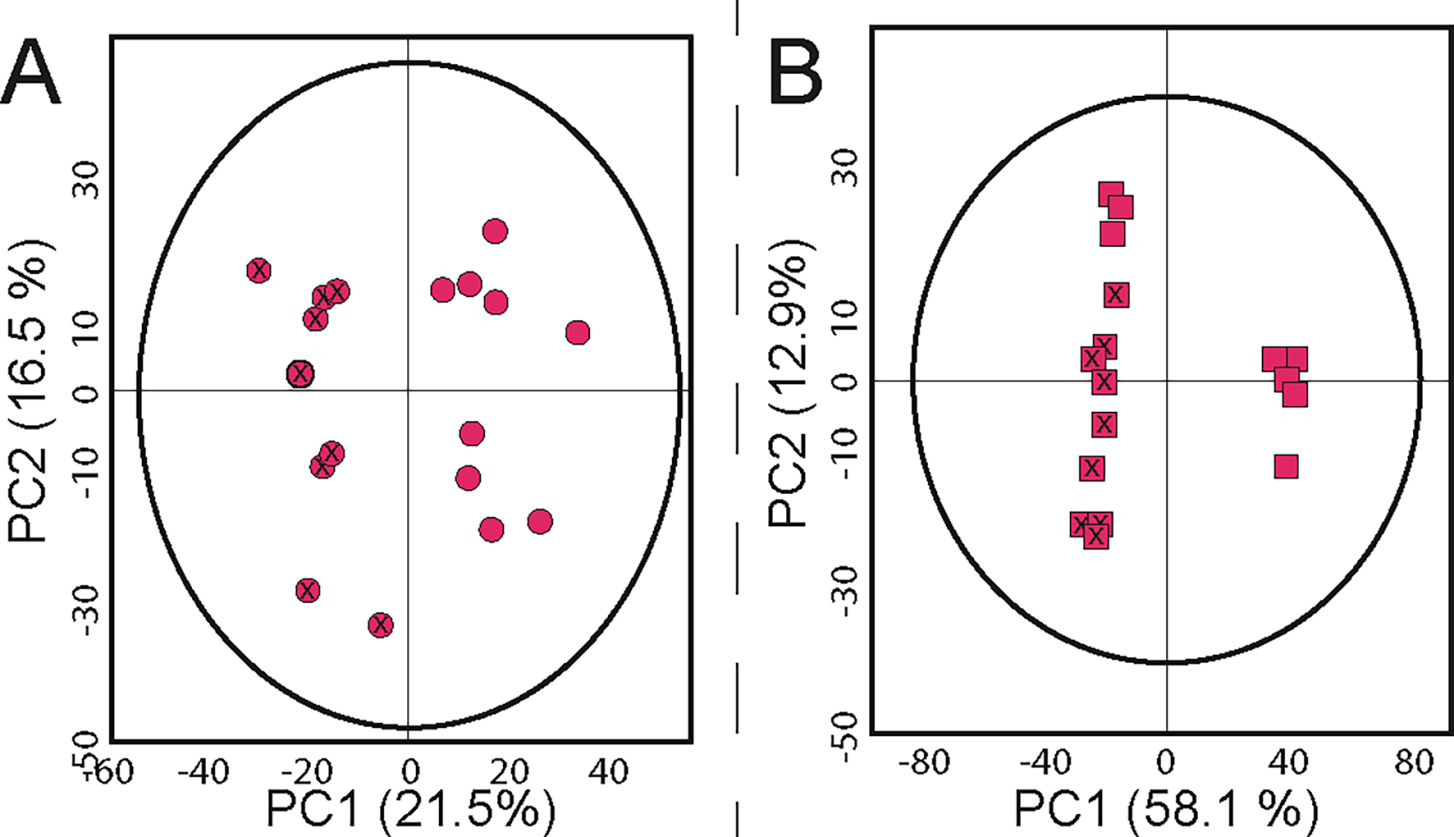
Principal component analysis of the untargeted metabolomic tests for less-polar/apolar metabolites of femora, comparing an affected group with a control group using cortical and trabecular samples from human osteoarchaeological remains in a C18 column and a nanoflow-UHPLC-Orbitrap-HRMS ESI positive mode platform. (**A**) Cortical bone R^2^(cum):0.603, Q^2^(cum):0.212, N = 18, 1780 variables; (**B**) trabecular bone R^2^(cum):0.71, Q^2^(cum):0.64. N = 18, 1623. X-Dots represent affected individuals in cortical bone. Dots represent individuals from the control group in cortical bone. X-squares represent affected individuals in trabecular bone. Squares represent individuals from the control group in trabecular bone. Ellipses indicate 95% of confidence for the PCA model. The explained variances of selected principal components are shown in brackets.

The PCA models derived from both analytical platforms allowed a reduction of variables, which permitted interpretation of the variance between the metabolomic profiles of the compared HSR groups. The explained variation for all models presented optimal predictive accuracy, except for the model corresponding to the TB in the C18-TOF platform, which presented a decreased accuracy with respect the other models. Nevertheless, statistical R^2^ and Q^2^ values validate all models. Figures 7 and 8 show the resultant PCA models for the measurement obtained with the HF-UPLC-IM-TOF-HRMS and the nano-UPLC-Orbitrap-HRMS, respectively.

After analysing the different statistical models for the metabolomic profile of CB and TB for both analytical platforms, it was evident that biological replicates presented low dispersion among observations for each individual. Keeping in mind that HSR material is irreplaceable, and scarce in the case of prehistoric material, researchers must evaluate the use of biological replicates for metabolomic studies, according to the availability of material for the study. Furthermore, the results highlight that the combination of different platforms and bone structures allows for the evaluation of a larger number and type of molecules present in HSR, which could make the identification of differences between a control and a studied condition possible. In general, PCA models reflect that for polar and less-polar/apolar metabolites, a slight-moderate separation between the control and affected group is present for CB and TB structures in the TOF platform. Meanwhile, an optimal separation between both groups is achieved through the Orbitrap platform for both bone structures.

Closer inspection of the different PCA models shows that details about health conditions in past populations could be explored through the combination of metabolomic data with physical osteological inspection. For example, measurement of the TB by the C18-UPLC-IM-TOFMS platform shows a separation between the affected individuals in two groups, distinct from the separation in respect to the control group. After a further inspection of the individuals by the osteoarchaeological team (see Supplementary Information, Table S4.), differences in bone colour and a reduction in bone mass, which may relate to pathology (e.g., osteoporosis/osteopenia) were observed. Individuals SK85 and SK209 both showed increased skeletal porosity, and SK1089 showed signs of osteoarthritis. In contrast, the other two individuals presented a higher bone weight, with signs of vitamin D deficiency (SK400) and spinal osteoarthritis (SK836). This indicates that metabolomic studies on HSR could offer new insight into the health conditions of past populations. Further dedicated testing on a larger sample focusing on a single condition would be the next logical step.

Figure S8 presents the volcano plots of the different compounds that have significantly decreased or increased in abundance (p < 0.05, Log2 FC > 2) between the control group and the affected group for all platforms studied here. Volcano plots confirm that metabolomic profiles in all bone structures and analytical platforms contain a series of compounds that are statistically similar between both groups. Nevertheless, differences in composition for polar and less-polar/apolar compounds can be detected for CB and TB structures.

Finally, in order to have a greater understanding of the potential of the information extracted from the metabolomic study of HSR, the different compounds found by the different analytical platforms were compared with databases and libraries to obtain their possible identification. A large list of hits was obtained. As there was no intention to explore the characterization or identification of single metabolites in the current study, the preference was to perform a mummichog approach to obtain a full list of compounds after the untargeted LC-HRMS measurements to evaluate the differences in the two studied groups. A complete list of the possible ID’s found for the different metabolites for each one of the analytical platforms is offered within the Supplementary Information (see Supplementary Information Data S1–S6). Table 1 presents the different pathways affected within both groups after performing the mummichog approach on the different data sets from three main libraries (i.e., Homo sapiens (human) [Kyoto Encyclopedia of Genes and Genomes, KEGG], Homo sapiens (human) [BioCyc], and Location-based Metabolite Sets (human)). To improve the quality of the operation, the polar and less-polar/apolar metabolites detected by the TOF-HRMS platform were combined as a single list for the mummichog analysis, as the algorithm sensitivity is directly proportional to the number of compounds in the input list.

**Table 1:** Mummichog pathways in HSR metabolomic study.

Bone structure and analytical platform	Mummichog pathways								
	Homo sapiens (human) [KEGG]			Homo sapiens (human) [BioCyc]			Location-based Metabolite Sets (human)		
	No. of pathways	Significant pathways*	Top three most significant pathway (Fisher exact p-value for the pathway)	No. of pathways	Significant pathways*	Most significant pathway	No. of pathways	Significant pathways*	Most significant pathway
CB TOF^+^	13	1	Taurine and hypotaurine metabolism (0.037905) Glycine, serine and threonine metabolism (0.19737) Sphingolipid metabolism (0.19737)	6	0	Glutathione biosynthesis (0.22581) Histamine degradation (0.53861) leukotriene biosynthesis (0.64485)	16	0	Fibroblasts (0.18126) Nerve Cells (0.2613) Neuron (0.2613)
TB TOF^+^	6	2	Pyrimidine metabolism (0.10349) Glycosaminoglycan degradation (0.10349) Drug metabolism—other enzymes (0.15162)	13	9	Cardiolipin biosynthesis II (0.064286) choline biosynthesis III (0.064286) phosphatidylcholine biosynthesis I (0.064286)	5	0	Spleen (0.15852) Epidermis (0.23069) Placenta (0.30785)
CB + TB TOF^+^	5	2	Sphingolipid metabolism (0.069364) Nicotinate and nicotinamide metabolism (0.069364) Pyrimidine metabolism (0.19504)	17	13	lipoate salvage I (0.099379) pregnenolone biosynthesis (0.099379) cardiolipin biosynthesis II (0.099379)	12	1	Skeletal Muscle (0.085996) Muscle (0.12594) Placenta (0.282)
CB Orbitrap^++^	29	2	Steroid hormone biosynthesis (0.0022601) Linoleic acid metabolism (0.046048) alpha-Linolenic acid metabolism (0.21629)	41	6	Androgen biosynthesis (0.0085819) Estrogen biosynthesis (0.0085819) 4-hydroxybenzoate biosynthesis (0.022462)	45	5	Gonad (0.014549) Endoplasmic Reticulum (0.032417) Adrenal Gland (0.033061)
TB Orbitrap^++^	43	1	Steroid hormone biosynthesis (0.00047033) Steroid biosynthesis (0.193) Retinol metabolism (0.36613)	76	0	Zymosterol biosynthesis (0.42954) Noradrenaline and adrenaline degradation (0.42954) Prostanoid biosynthesis (0.48528)	48	10	Muscle (0.0034873) Gonad (0.0054478) Mitochondria (0.0054478)
CB + TB Orbitrap^++^	45	2	Steroid hormone biosynthesis (0.00038518) Arachidonic acid metabolism (0.068272) Retinol metabolism (0.14761)	73	0	Prostanoid biosynthesis (0.44568) Zymosterol biosynthesis (0.44568) Androgen biosynthesis (0.51054)	48	3	Kidney (0.0012333) Liver (0.0029415) Gonad (0.047184)

Affected pathways found in the comparison of metabolites from the control and affected groups of HSR for cortical (CB), trabecular (TB), and combined bone structures after LC-HRMS measurements using HF-UPLC-IM-TOF-HRMS and nano-UHPLC-Orbitrap-HRMS platforms.

^+^Pathways explored combining C18 and HILIC results.

^++^Pathways explored using C18 results.

*Fisher exact p-value p < 0.1.

Mummichog comparisons revealed that impacted pathways detected vary depending on the bone structure (i.e., CB or TB). This further supports our previous finding that the nature/number of metabolites are not identical in each structure. When datasets from each bone structure are combined in a single matrix to perform the mummichog approach, results show that produced pathways are not the direct addition of the pathways found for each separated structure. Instead, when the list of compounds is extended for the analysis, some pathways are preserved, at the time that new affected pathways are evidenced. Furthermore, mummichog analysis shows that the total number and type of affected pathways found for each analytical platform in a single bone structure were different, this being ascribable to the number of compounds detected by one or other analytical platform. The orbitrap-HRMS platform presented a higher number of detected compounds, and a higher number of pathways could be identified. It is important to highlight that this difference is not due to the nature of the HSR, but to the technical capabilities of the instrumentation used for the metabolomic assay.

## Conclusions

For the first time, human osteoarchaeological remains were used as a case study in a metabolomic approach to confirm the presence of biological polar and less polar/apolar metabolites by LC-HRMS. Rigorous protocols for both microsampling and extraction were developed to decrease the total mass of material required, which, combined with appropriate quality assurance processes and bioinformatics, allowed characterisation of differences in the metabolomic profiles in the resultant spectrometric data of different individuals tested.

Overall, results confirm that metabolomic information can be extracted from HSR, and that the information can vary between bone structures. Contrary to what is traditionally considered within archaeological science, here we demonstrate that human osteoarchaeological material cannot be thought of as a single “tissue”, but as a combination of structures, each containing different metabolomic information that can be investigated through different analytical platforms to extract information for the interpretation of conditions in previous populations. To obtain results that can be used to interpret the biological condition evaluated in the metabolomic assay, our work showed the importance of the experimental design in decreasing the variability in the data by controlling for the intrinsic confounders of the samples. Tissue type (i.e., CB, PB, TB, or a mix), skeletal element used (i.e., femora, humeri, tibiae, ribs, 1st and 2nd metatarsals, etc.), and post-excavation status of the remains (i.e., dry cleaned, wet cleaned, etc.) are some of the factors to be considered and controlled for, as these can directly impact the metabolomic results.

This research shows for the first time that HSR material are a feasible source of information for untargeted metabolomic studies. Sampling and extraction methods presented allowed us to obtain hundreds of different polar and less polar/apolar molecular features associated with the biological composition of the individuals studied through LC-HRMS measurements. Methods and analytical improvements can be achieved for specific molecules, or by considering other analytical platforms such as the nuclear magnetic resonance, and gas chromatography, among others.

After performing a pilot metabolomic assay of a controlled *vs.* affected group of individuals on two different analytical platforms, we show that metabolomic differences between groups were spotted in polar and less polar/apolar metabolites. This demonstrates that HSR have the potential to be used in metabolomic studies to obtain biomarkers, pathways, or other types of information relevant to the human past.

Results presented here show an example of the possible untargeted metabolomic data that can be obtained from HSR. It is expected that this will open up interest of different scholars who might want to use HSR matrices for new metabolomic studies, and at the same time, undertake further studies to evaluate the analytical differences obtained not only through comparison of different mass analysers but also for different separation methods and equipment.

## Supplementary Information

Supplementary Information 1.

Supplementary Information 2.

Supplementary Information 3.

Supplementary Information 4.

Supplementary Information 5.

Supplementary Information 6.

Supplementary Information 7.

## Data Availability

All data needed to evaluate the conclusions in the paper are present in the paper and/or the Supplementary Information. RAW MS data used in this study can be consulted at MetaboLights www.ebi.ac.uk/metabolights/MTBLS4437. Further data about the extraction method is available under request to corresponding author.
