# Supplementary material for: Examination of human osteoarchaeological remains as a feasible source of polar and apolar metabolites to study past conditions
Source: Sci Rep. 2023 Jan 13;13:696. doi: 10.1038/s41598-023-27401-0 (PMC9839756; doi:10.1038/s41598-023-27401-0)
Supplement: Supplementary file 1 — Supplementary Information 1. [file 41598_2023_27401_MOESM1_ESM.docx]

Supplementary Information for

**Examination of Human Osteoarchaeological Remains as a Feasible Source of Polar and Apolar Metabolites to Study Past Conditions.**

Diego Badillo-Sanchez^1^, Maria Serrano Ruber^1^, Anna M. Davies-Barrett^1^, Jatinderpal K. Sandhu^2,3^, Donald J.L. Jones^2,3^, Martin Hansen^4^, Sarah A. Inskip^1,*^

*Corresponding authors: s.inskip@leicester.ac.uk

**This file includes:**

Supplementary Text

Figures S1 to S8.

Tables S1 to S4.

**Other Supplementary Materials for this manuscript include the following:**

Data S1 to S6

**Supplementary Text**

**Variable pressure scanning electron microscopy coupled with energy dispersive X-ray spectroscopy**

A variable pressure scanning electron microscope (VP-SEM Carl Zeiss Microsystems GmbH), coupled with an energy dispersive X-ray spectroscopy detector (EDS XFlash 5010 detector - Bruker, USA; 129eV Energy Resolution an Mn/Kα FWHM) was used to characterize the topography, as well as to obtain the elemental information from the different cortical and trabecular micro-samples from the human osteoarchaeological remains. Operating conditions were as follows: voltage 20kV, vacuum = 40Pa; WD = 8.5 mm. Neither gold nor carbon coating was required.

**Metabolite putative annotation**

Putative annotation on the compounds found by the High flow-UPLC‑ IM-TOF‑HRMS measurements on C18 and HILIC experiments was performed using the data identification window of the Progenesis QI software. Data was compared considering all potential matches using the following libraries: 1) METLIN MS/MS library 2017 for Progenesis QI plugin with a 3-ppm precursor and fragment tolerance for H=0-150; C=0-100; N=0-10; O=0-30; F=0-4; Mg, P, S, Cl, Br, I =0-2. 2) LipidBlast library plugin with a 3-ppm precursor tolerance and 10-ppm fragment tolerance. 3) Metabolic Profiling CCS Library search plugin with a 3-ppm precursor tolerance, 10 ppm fragment tolerance, and 10% CCS tolerance. Putative annotation on the compounds found by the nano -UHPLC-Orbitrap-HRMS instrument on C18 were subject to a MS/MS identification process combining the MS1 and MS/MS files in the Compound discover software following the ID using online databases and MZlogic.

**Detail analysis of PCA’s from Untargeted metabolomic analysis of HSR, comparing a control versus a condition group by the use of two different HRMS platforms**

Figure 7A. shows the PCA model for polar metabolites from CB measured by the TOF platform. This PCA model shows that observations presented the tendency to be aligned along PC2, with the affected group shifted towards more negative values in the PC1 and more positive values in the PC2 than observations from the control group. The exception was for three replicates from individual SK400, which presented a significant positive displacement on PC1, separating it from the other observations. Observations from the control group can be split into two clusters, one composed of individuals SK3, SK5, SK7, and SK8, with positive PC2 values; and a second composed of SK1, SK4, and SK6, with negative PC1 values. Triplicates from SK3 presented a dispersion, with one replicate as a statistical outlier (SK3_1). Observations from the affected group are split into three groups, one composed of SK836, SK209, and SK1089, located in positive values of PC2; a second group composed of SK85, with negative values of PC2; and a third group composed of SK400, with positive PC1 and PC2 values. Triplicates from the affected group clustered together. This result agrees with our previous observations, whereby polar metabolites of cortical bone do not present higher variance. Figure 7B. shows the PCA model for the less-polar/apolar metabolites of CB measured by the TOF platform. The PCA model shows a clustering of the observations of the control group around the centre of the PCA model, with an optimal separation with respect to the affected group. Observations of the affected group can be split into three groups with respect to the control group, one composed of SK209, SK85, and SK1089, located towards more positive PC2; a second composed of SK836, located to more negative PC1 and more positive PC2 values; and a third composed of SK400, located towards more positive PC1 values. Triplicates from the control and affected groups present lower dispersion, except for SK3_1, which presents an outlier behaviour, as was found for the polar measurement.

Figure 7C. shows the PCA model for polar metabolites from TB measured by the TOF platform. The PCA model shows that observations from the control group presented an optimal separation from those in the affected group, which presented a tendency to have more positive PC2 values than the control group. Control group observations can be split into three groups, one composed of SK1 and SK3, with negative PC2 values; a second composed of SK4 and SK2, with positive PC1 values; and a third composed of SK5, with negative PC1 and positive PC2 values. Observations from the affected group are also split into three, one main group composed of SK1089, SK 209, and SK836, with negative PC1 and positive PC2 values; a second composed of SK400, with positive PC1 and PC2 values; and a third composed of SK85, with negative PC1 and PC2 values. Triplicates for both the control and affected group present low variance clustering close to one another. Figure 7D. shows the PCA model for less-polar/apolar metabolites from CB measured by the TOF platform. The PCA model shows that observations from control individuals present a tendency for positive PC1 and PC2 values, being separated from the observations of the affected group. The control group observations from SK1, SK3, and SK4 cluster together, meanwhile SK2 is negatively displaced relative to PC1 and PC2 values. SK5 presents an outlier behaviour. The affected group can be split into two, one group composed of individuals SK209, SK85, and SK1089, with negative PC1 and positive PC2 values; and a second group composed of individuals SK400 and SK836, located towards negative PC2 values.

Figure 8A. shows the PCA model for less-polar/apolar metabolites from CB as measured by the Orbitrap platform. This shows that observations from the control group are well separated from those of the affected group. Control group observations can be split into two groups, one composed of SK3, SK4, and SK5, located towards positive PC1 and PC2 values; and a second group composed of SK1 and SK2, located towards positive PC1 and negative PC2 values. Triplicates of the control group present some variance, but the overall tendency is preserved. Observations from the affected group can be split into three groups, one composed of SK400, SK1089, and SK85, with negative PC1 and positive PC2 values; a second composed of SK 209, with negative PC1 and PC2 values; and a third composed of SK836, located towards negative PC2 values. Figure 8B. shows the PCA model for less-polar/apolar metabolites from TB measured by the Orbitrap platform. The PCA model shows that observations from the control group are easily identified as a separate group apart from the affected group. Observations from the control group are split into two, one cluster composed of SK1, SK2, SK3 and one replicate of SK4, located at positive PC1 values; and a second group composed of SK5 and two replicated from SK42, located towards negative PC1 and positive PC2 values. Observations from the affected group are clustered in negative PC1 and scatter through PC2 values, with SK85, SK400, and SK836 with positive PC2 values, and SK1089 and SK209 with negative values. In general, triplicates present low dispersion, presenting similar tendencies among them for both control and affected groups.


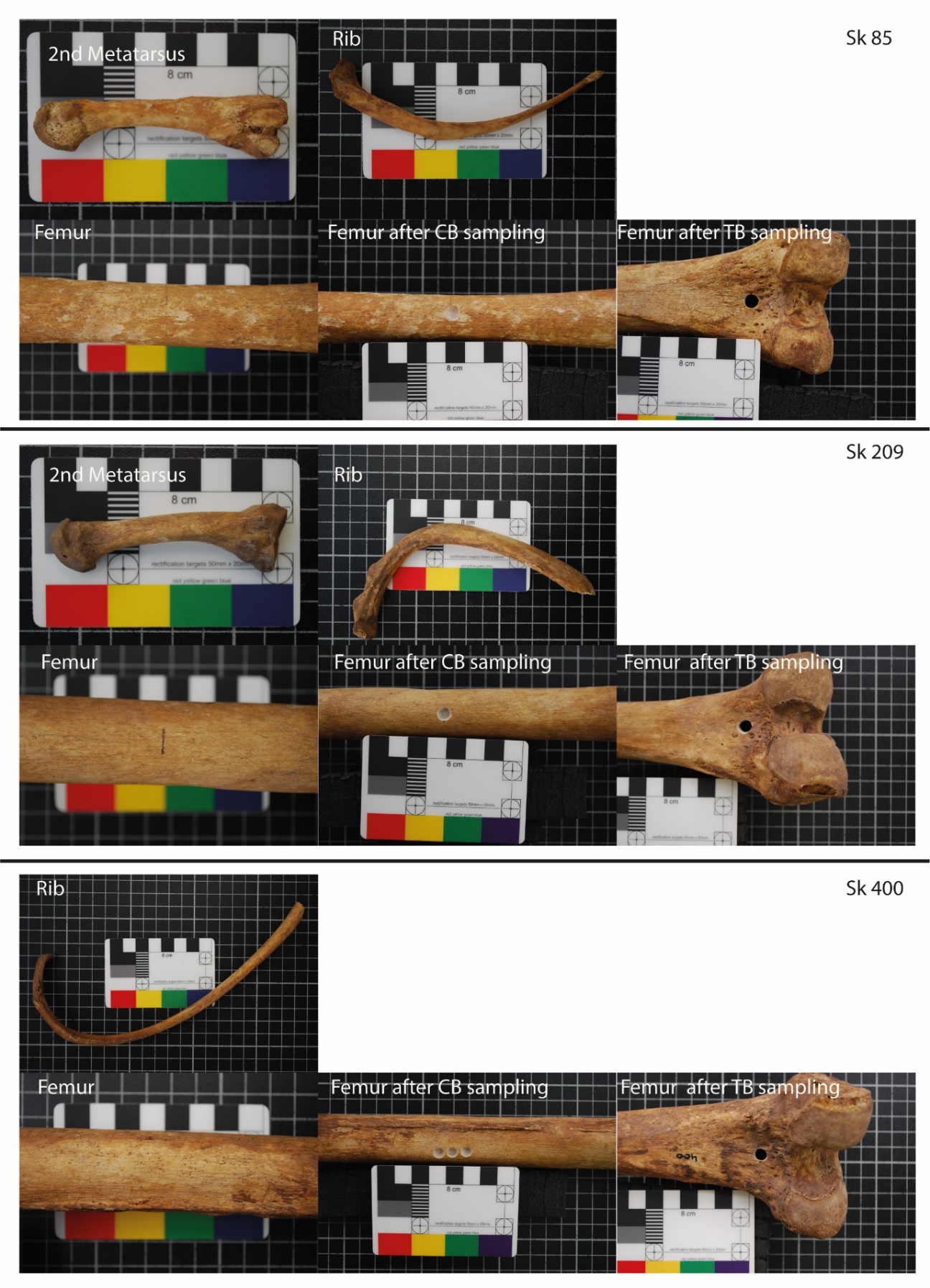


**Figure S1.** Photographs of the different articulated human skeletal remains from Coventry (UK).


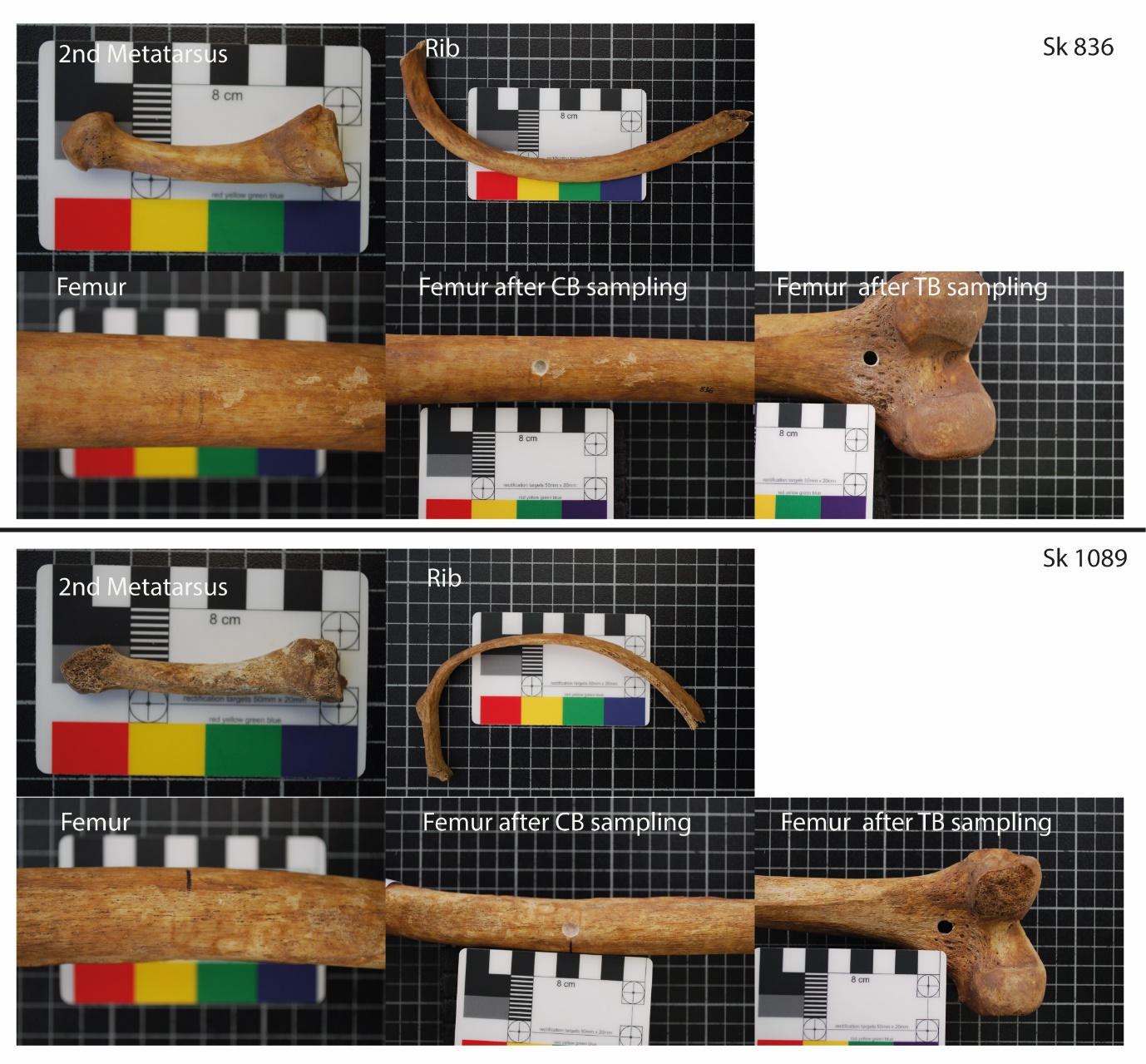


**Figure S1.** (continuation) Photographs of the different articulated human skeletal remains from Coventry (UK). Photographs of the different skeletal elements selected from the articulated HSR from Coventry, UK, used in the metabolomic studies to obtain both cortical (CB) and trabecular (TB) micro-samples. Detail of resultant perforations after the extraction of CB and TB in a femur are shown.


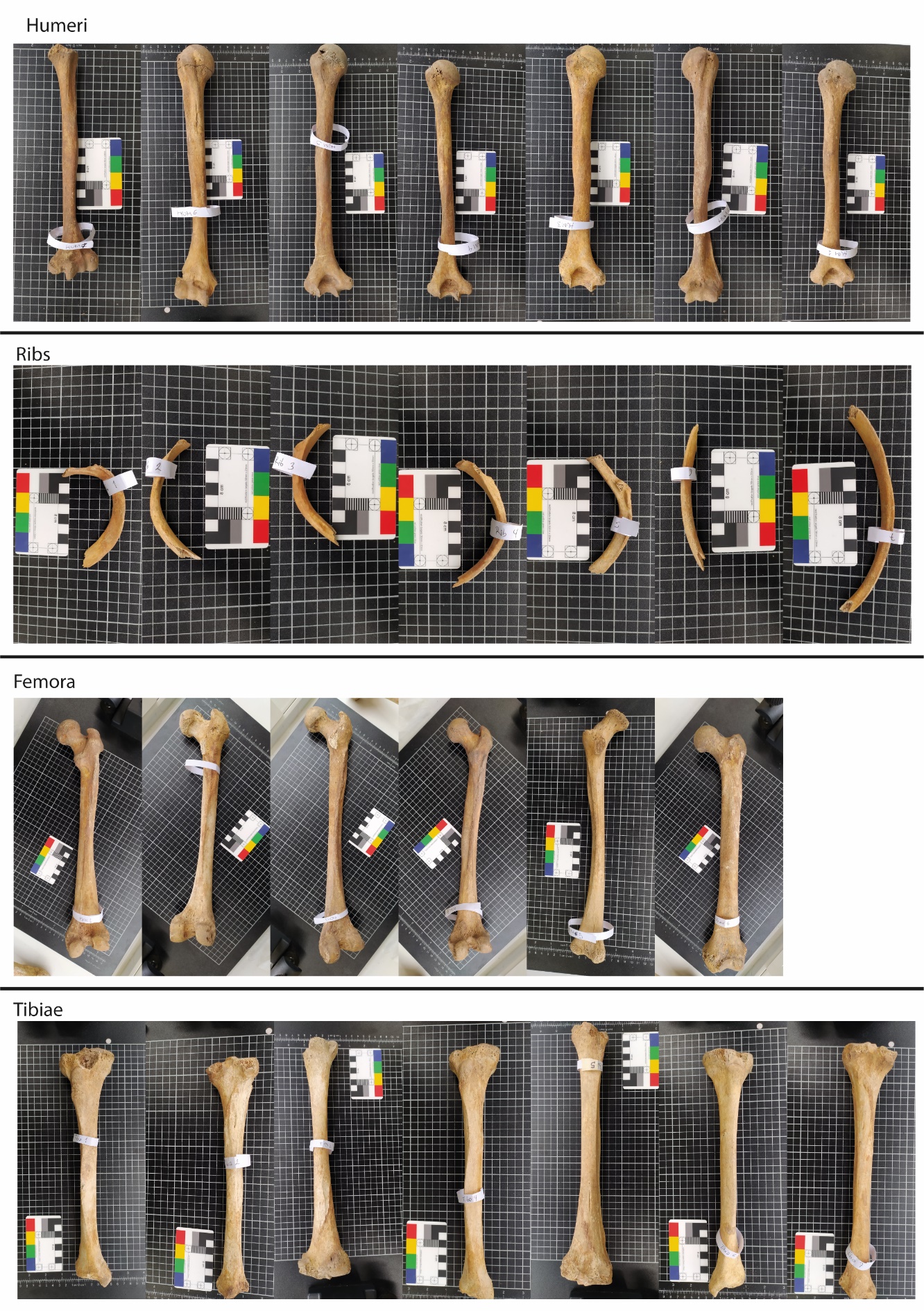


**Figure S2.** Photographs of the different disarticulated human skeletal remains from Cambridge (UK).


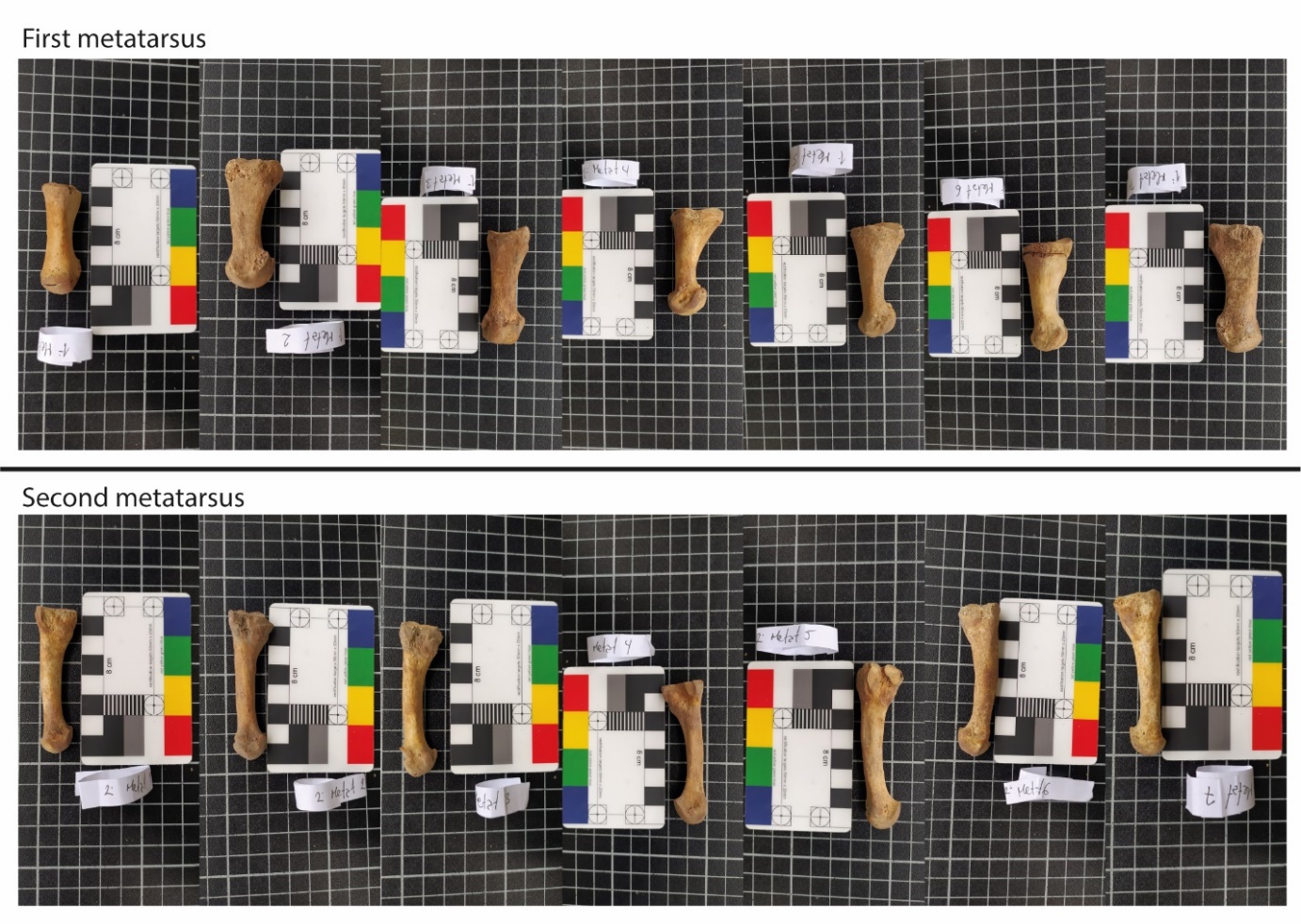


**Figure S2.** (continuation) Photographs of the different disarticulated human skeletal remains from Cambridge (UK). Photographs of the different skeletal elements selected (humeri, tibiae, femora, ribs, first and second metatarsals) from the disarticulated HSR from Cambridge, UK, used in the metabolomic studies to obtain both cortical (CB) and trabecular (TB) micro-samples.


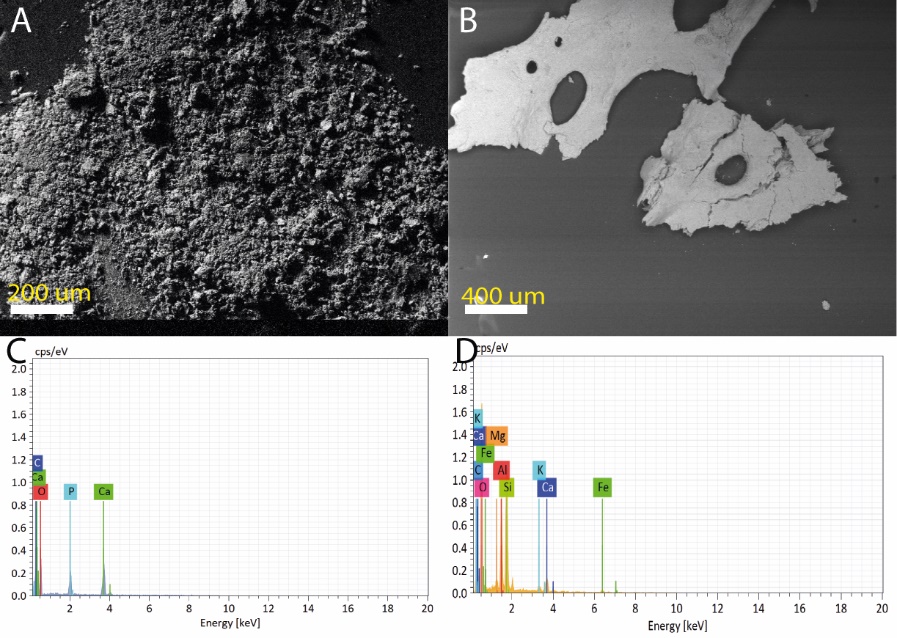


**Figure S3**. Elemental composition and microphotography for cortical and trabecular bone microsamples. SEM micro images and EDS elemental composition spectra measured for both cortical and trabecular bone micro-samples after the sampling drilling process in Sk86. A) Cortical bone microimage; B) Trabecular bone microimage; C) Punctual EDS spectrum for cortical bone; D) Punctual EDS spectrum for trabecular bone.


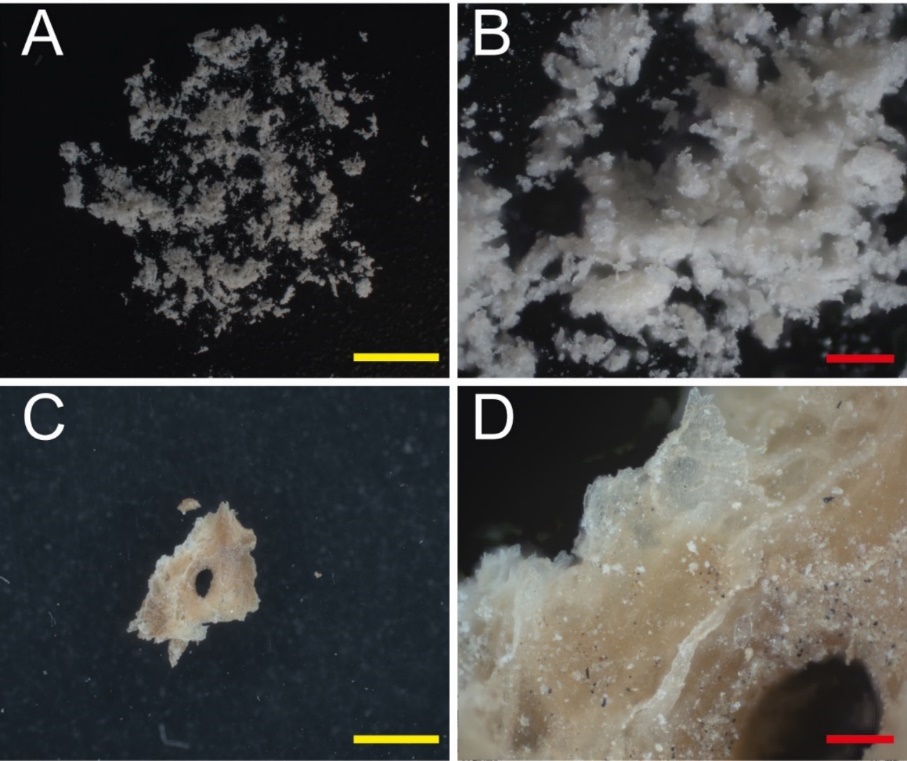


**Figure S4.** Cortical and trabecular bone micro-samples. Microphotographs of the bone micro-samples in Individual SK400. (A, B) Cortical bone; (C, D) trabecular bone. Red line indicates a scale of 100 µm in length and yellow line a scale of 1 mm in length.

**
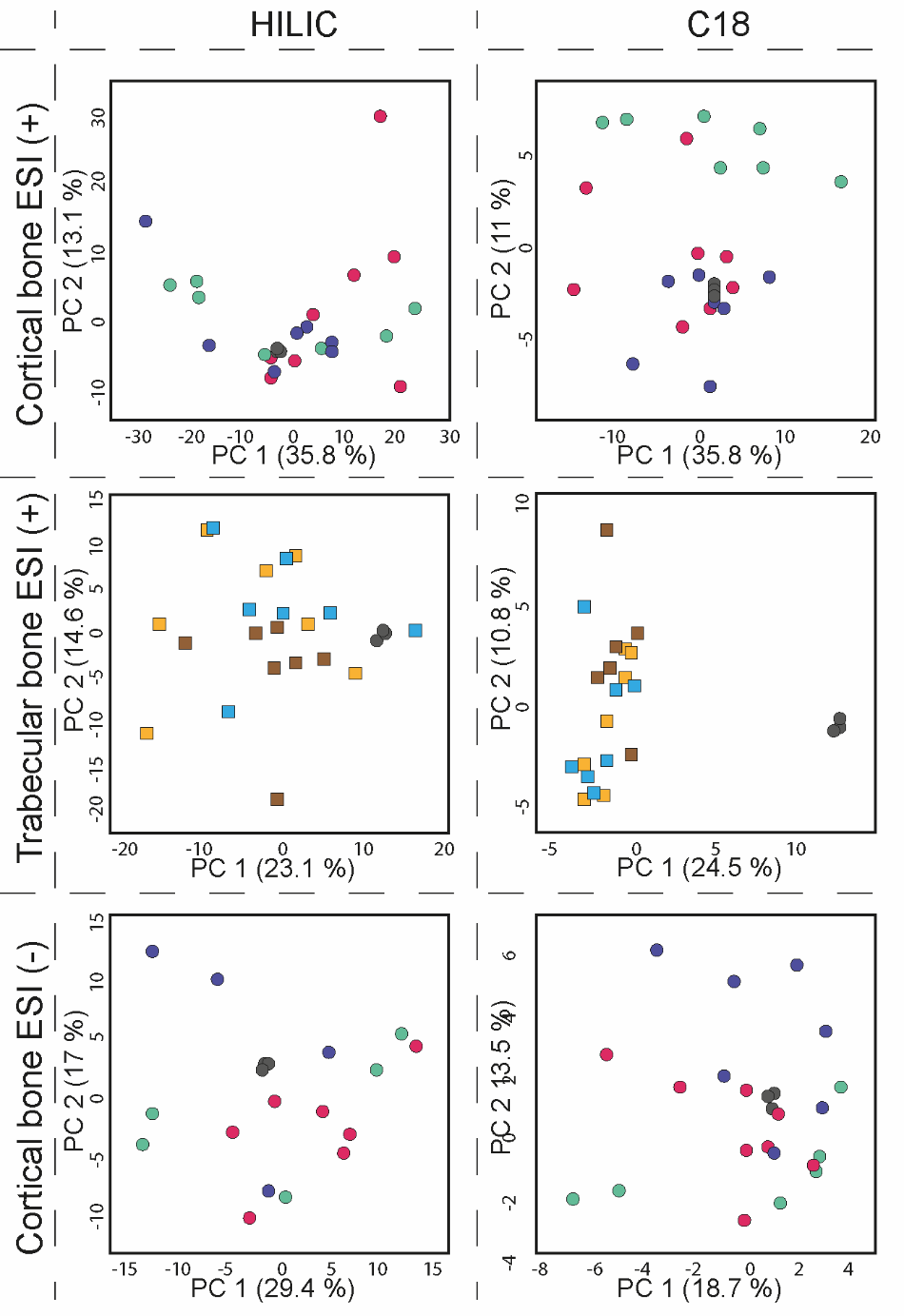
**

**Figure S5.** Principal component analysis for the untargeted metabolomic test using cortical (dots) and trabecular bone (squares) from different skeletal elements. PCA score plot with pooled QC samples. Observations are colored according to each skeletal element: femur, red; humerus, green; tibia, purple;1^st^ metatarsus, yellow; 2^nd^ metatarsus, blue; rib, brown. Black dots represent pooled QC samples.

––––
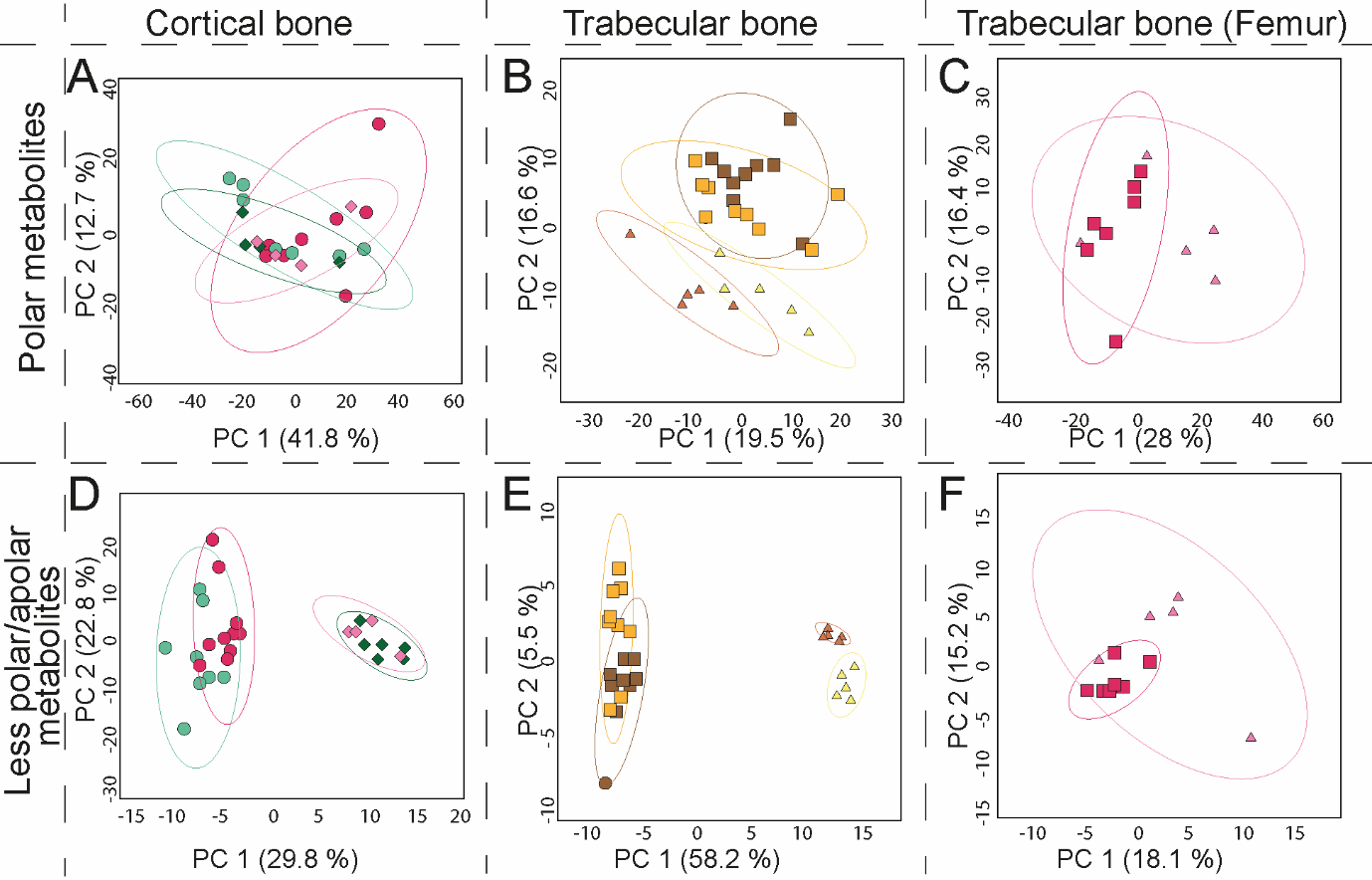


**Figure S6.** Principal component analysis for the untargeted metabolomic tests using cortical (CB) and trabecular bone (TB) from different human skeletal elements before and after water cleaning. Score plots for the different metabolites extracted and measured through HF-UPLC-IM-TOF-HRMS in ESI (positive mode) for cortical samples –from femora and humeri– and trabecular samples –from first metatarsals, ribs, and femora– in disarticulated human osteoarchaeological remains before and after being subject to water cleaning. Data matrices were subject to data cleaning, normalized, transformed, and scaled (see Materials and Methods section). (A) Polar metabolites in cortical bone (HILIC) N=29, 1629 variables; (B) Polar metabolites in trabecular bone (HILIC) N=28, 1397 variables; (C) Polar metabolites in trabecular bone from femora (HILIC) N=12, 1397 variables; (D) Less-polar/apolar metabolites in cortical bone (C18) N=29, 665 variables; (E) Less-polar/apolar metabolites in trabecular bone (C18) N=28, 312 variables; (F) Less-polar/apolar metabolites in trabecular bone from femora (C18) N=12, 312 variables. Ellipses indicate 95% of confidence for each group. The explained variances of selected principal components are shown in brackets. Observations are colored according to each skeletal element: femur, red; humerus, green; tibia, purple;1^st^ metatarsus, yellow; 2^nd^ metatarsus, blue; rib, brown. Shapes of the observations represents: dots, No washed CB; squares, No washed TB; diamonds, washed CB; triangles, washed TB.

**
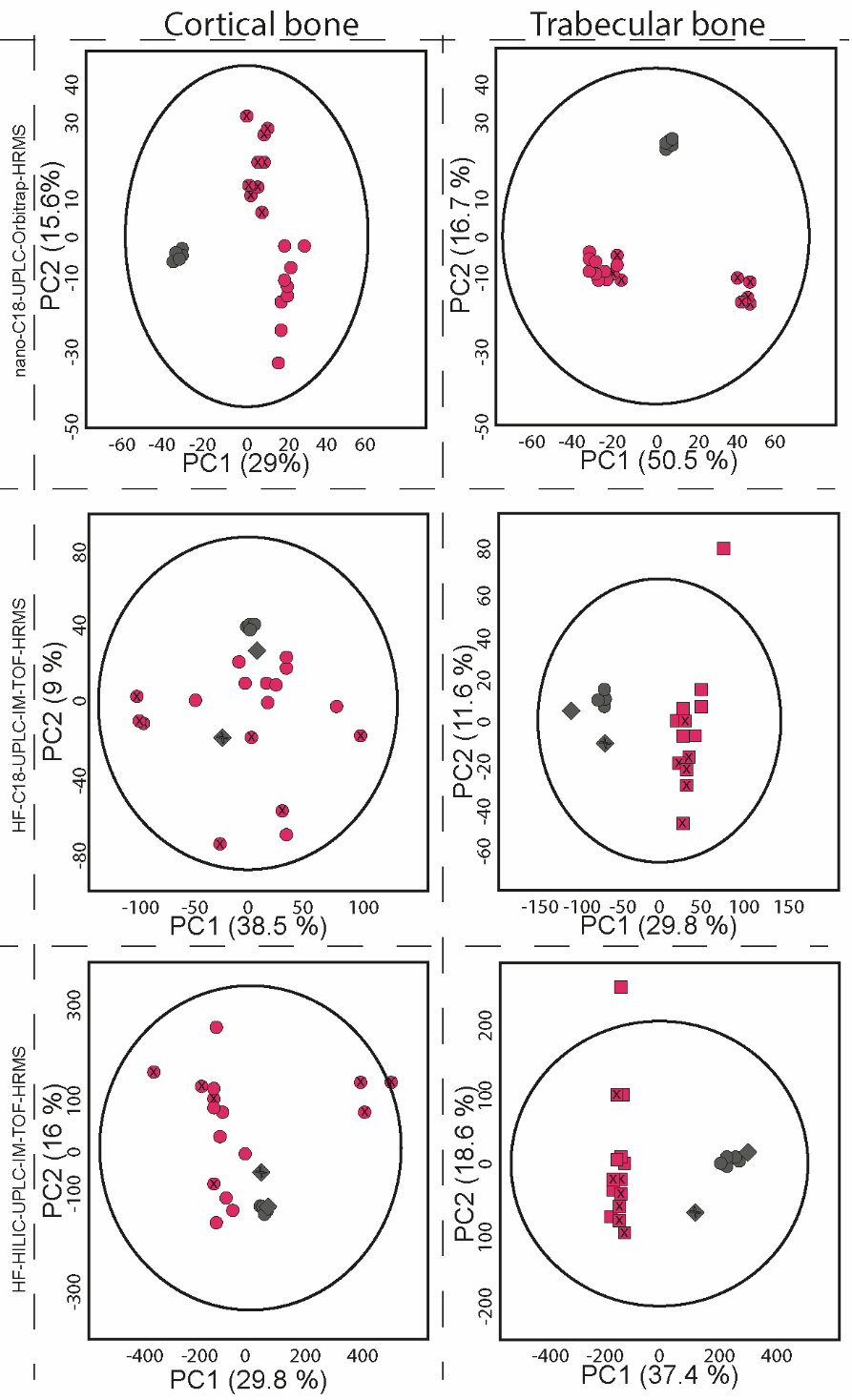
**

**Figure S7.** Principal component analysis for the untargeted metabolomic test of femora, comparing an affected group with a control group using cortical and trabecular samples from human osteoarchaeological remains. PCA score plot with pooled QC samples. X-Dots represent affected individuals in cortical bone. Dots represent individuals from the control group in cortical bone. X-squares represent affected individuals in trabecular bone. Squares represent individuals from the control group in trabecular bone. Black dots represent pooled QC samples. Black diamonds represent QC samples for each tested group. The explained variances of selected principal components are shown in brackets


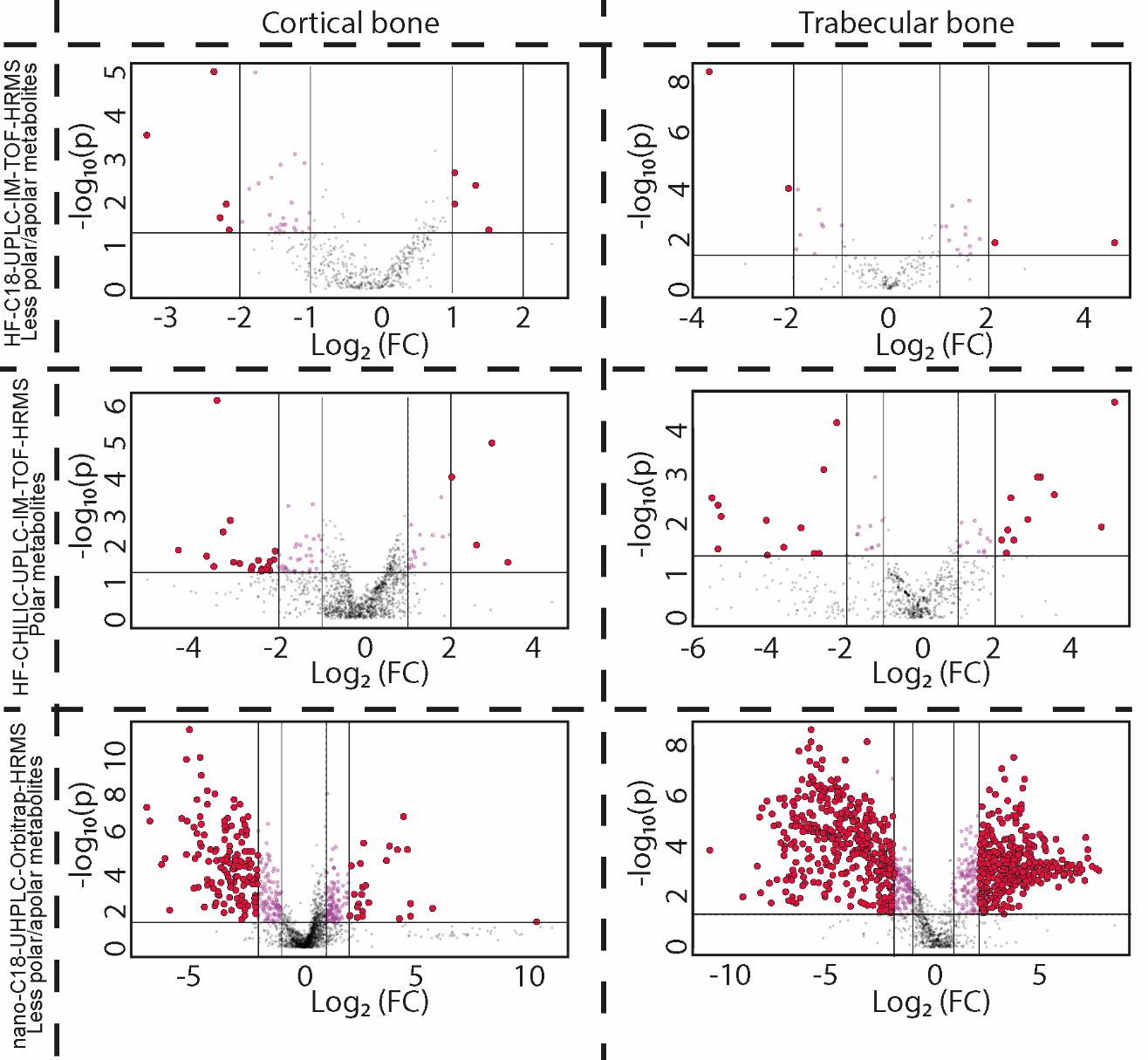


**Figure S8.** Volcano plots for the comparison of the metabolomic profiles in HSR. Volcano plots were built using the metabolomic profiles obtained for control and affected groups after the LC-HRMS measurement. Left panels show comparison of metabolomic profiles from cortical structures. Right panels present the comparison of metabolomic profiles from trabecular structures. Top panels present results for polar metabolites after measurement with the HF-UPLC-IM-TOF-HRMS platform. Central panels present results for less-polar/apolar metabolites after measurement with the HF-UPLC-IM-TOF-HRMS platform. Bottom panels present results for less-polar/apolar metabolites after measurement with the nano-UPLC-Orbitrap-HRMS platform. Red dots represent compounds with significantly decreased or increased value (p<0.05, and a Log_2_ FC>2) between the affected and control groups. Black dots represent metabolites with insignificant variance between groups. Purple dots represent compounds with significantly decreased or increased value (p<0.05, and a Log_2_ FC>1<2).

| Provenance | ID | Mass/g | Length/cm | Perimeter/cm | Diameter/mm | Length/mass ratio |
| --- | --- | --- | --- | --- | --- | --- |
| Cambridge, UK | SK1_Rib | 7.04 | 15.4 | 3.6 | 11.19 | 2.19 |
|  | SK2_Rib | 5.35 | 12.5 | 3.9 | 11.86 | 2.34 |
|  | SK3_Rib | 7.2 | 12.7 | 4.1 | 8.22 | 1.76 |
|  | SK4_Rib | 6.69 | 14 | 4.4 | 14.56 | 2.09 |
|  | SK5_Rib | 6.95 | 13.1 | 3.9 | 10.25 | 1.88 |
|  | SK6_Rib | 6.69 | 11.8 | 3.9 | 10.05 | 1.76 |
|  | SK7_Rib | 8.82 | 18.9 | 3.5 | 10.48 | 2.14 |
|  | SK1_Humerus | 98.91 | 30 | 6.8 | 20.19 | 0.30 |
|  | SK2_Humerus | 83.97 | 29 | 6.2 | 20.63 | 0.35 |
|  | SK3_Humerus | 133.72 | 31.7 | 7.3 | 23.43 | 0.24 |
|  | SK4_Humerus | 84.65 | 30.2 | 6 | 17.58 | 0.36 |
|  | SK5_Humerus | 134.23 | 33 | 7 | 20.28 | 0.25 |
|  | SK6_Humerus | 131.53 | 34.5 | 7.6 | 21.96 | 0.26 |
|  | SK7_Humerus | 64.59 | 26.5 | 6 | 17.53 | 0.41 |
|  | SK1_Femur | 286.17 | 42.9 | 9 | 21.84 | 0.15 |
|  | SK2_Femur | 305.68 | 40.6 | 8.8 | 26.06 | 0.13 |
|  | SK3_Femur | 357.89 | 46.2 | 8.9 | 25.86 | 0.13 |
|  | SK4_Femur | 268.45 | 41.4 | 9 | 30.07 | 0.15 |
|  | SK5_Femur | 126.48 | 35.6 | 6.4 | 19.1 | 0.28 |
|  | SK6_Femur | 391.31 | 45.5 | 9.8 | 32.96 | 0.12 |
|  | SK7_Femur | 366.99 | 43 | 9.1 | 29.34 | 0.12 |
|  | SK8_Femur | 255.57 | 42 | 8.1 | 24.72 | 0.16 |
|  | SK1_Tibia | 168.36 | 33 | 8 | 21.67 | 0.20 |
|  | SK2_Tibia | 116.38 | 30.2 | 7 | 17.74 | 0.26 |
|  | SK3_Tibia | 231.33 | 36 | 8.9 | 24.56 | 0.16 |
|  | SK4_Tibia | 109.09 | 30.5 | 6.8 | 15.53 | 0.28 |
|  | SK5_Tibia | 192.58 | 37.5 | 8.8 | 21.76 | 0.19 |
|  | SK6_Tibia | 104.16 | 32.3 | 7.9 | 20.17 | 0.31 |
|  | SK7_Tibia | 154.23 | 33.5 | 7.6 | 20.19 | 0.22 |
|  | SK1_1Metatarsus | 3.94 | 4.9 | 4.4 | 11.67 | 1.24 |
|  | SK2_1Metatarsus | 8.07 | 6.1 | 5.3 | 14.12 | 0.76 |
|  | SK3_1Metatarsus | 7.18 | 6.6 | 4.6 | 11.72 | 0.92 |
|  | SK4_1Metatarsus | 7.6 | 5.7 | 4.5 | 11.83 | 0.75 |
|  | SK5_1Metatarsus | 7.21 | 6.4 | 4.7 | 12.02 | 0.89 |
|  | SK6_1Metatarsus | 8.31 | 6.2 | 5.3 | 13.06 | 0.75 |
|  | SK7_1Metatarsus | 9.24 | 6.4 | 5.6 | 14.9 | 0.69 |
|  | SK1_2Metatarsus | 5.5 | 6.8 | 3.6 | 8.93 | 1.24 |
|  | SK2_2Metatarsus | 4.91 | 7.3 | 3.5 | 8.32 | 1.49 |
|  | SK3_2Metatarsus | 3.52 | 7.2 | 3.5 | 8.06 | 2.05 |
|  | SK4_2Metatarsus | 4.92 | 7 | 3.4 | 6.65 | 1.42 |
|  | SK5_2Metatarsus | 6.95 | 7.7 | 3.4 | 8.95 | 1.11 |
|  | SK6_2Metatarsus | 6.56 | 7.7 | 3.6 | 10.4 | 1.17 |
|  | SK7_2Metatarsus | 5.2 | 6.7 | 3.5 | 8.78 | 1.29 |
| Coventry, UK | SK85_Rib | 5.47 | 13.6 | 3.1 | 3.58 | 2.49 |
|  | SK209_Rib | 10.21 | 16.7 | 4.9 | 8.74 | 1.64 |
|  | SK400_Rib | 16.03 | 30.6 | 4 | 7.12 | 1.91 |
|  | SK836_Rib | 9.75 | 19 | 3.6 | 7.35 | 1.95 |
|  | SK1089_Rib | 5.94 | 20.4 | 3.1 | 3.19 | 3.43 |
|  | SK85_Femur | 312.36 | 46.4 | 8.9 | 26.82 | 0.15 |
|  | SK209_Femur | 334.98 | 47.5 | 9 | 27.19 | 0.14 |
|  | SK400_Femur | 404.8 | 49.6 | 9.7 | 31.51 | 0.12 |
|  | SK836_Femur | 391.9 | 46.8 | 9.4 | 28.66 | 0.12 |
|  | SK1089_Femur | 217.54 | 44.6 | 7.9 | 24.57 | 0.21 |
|  | SK85_2Metaarsus | 5.01 | 7.2 | 2.9 | 5.92 | 1.44 |
|  | SK209_2Metatarsus | 5.6 | 7.3 | 2.7 | 7.53 | 1.30 |
|  | SK836_2Metatarsus | 5.52 | 6.7 | 2.8 | 7.67 | 1.21 |
|  | SK1089_2Metatarsus | 3.93 | 7.1 | 3.1 | 7.26 | 1.81 |

**Table S1.** List of human skeletal remains used to obtain the trabecular and cortical bone microsamples for the different metabolomic experiments.

| Column (Ionization) | Total molecular features | CB (*) | TB (*) |
| --- | --- | --- | --- |
| C18 (ESI+) | RAW | 3033 | 3352 |
|  | Cleaned | 665 (22%) | 312 (9%) |
| C18 (ESI-) | RAW | 1154 | 1742 |
|  | Cleaned | 154 (13%) | 243 (14%) |
| HILIC (ESI+) | RAW | 5258 | 6466 |
|  | Cleaned | 1629 (31%) | 1395 (22%) |
| HILIC (ESI-) | RAW | 2503 | 4157 |
|  | Cleaned | 703 (28%) | 471 (11%) |

**Table S2.** List of molecular features obtained after UPLC -IM-TOF-HRMS, before and after data processing for the different separation and ionization methods employed in the metabolomic study of cortical (CB) and trabecular bone (TB) structures of different disarticulated human osteoarchaeological remains to test variance of metabolomic profile in HSR material by using different skeletal elements and structures in an untargeted metabolomic study. ESI: Electrospray ionization; + positive mode; - negative mode; *Percentage of ions attributed to biological metabolites from the metabolomic test after data processing and cleaning.

| Method | | | C18 ESI+ IM-TOF | HILIC ESI+ IM-TOF | nano-C18 ESI+ Orbitrap |
| --- | --- | --- | --- | --- | --- |
| CB | Features | Raw | 1988 | 3849 | 4283 |
|  |  | Cleaned | **444** | **1283** | **1780** |
|  | Volcano | Up | 4 | 20 | 135 |
|  |  | Down | 30 | 55 | 236 |
| TB | Features | Raw | 1622 | 4038 | 4012 |
|  |  | Cleaned | **192** | **689** | **1623** |
|  | Volcano | Up | 14 | 23 | 485 |
|  |  | Down | 10 | 23 | 487 |

**Table S3.** Total of features detected for both cortical (CB) and trabecular bone (TB) microsamples, before and after the data treatment process, and total of features up- and down-regulated in the volcano plots for the metabolomic study of human skeletal remains in the untargeted metabolomic analysis of HSR, comparing a control versus a condition group by the use of two different HRMS platforms. ESI: Electrospray ionization; + positive mode.

| Femur ID | Medial Ante-Posterior width/mm | Medial Medio-Lateral width/mm | Medial Perimeter/cm | Mass/g |
| --- | --- | --- | --- | --- |
| SK400 | 29,96 | 32,2 | 9,7 | 404,8 |
| SK836 | 31,16 | 28,66 | 9,4 | 391,9 |
| SK1089 | 25,75 | 24,65 | 7,9 | 217,54 |
| SK209 | 30,91 | 27,15 | 9,0 | 334,98 |
| SK85 | 29,42 | 26,67 | 8,9 | 312,36 |

**Table S4.** Physical measurements of the femora of the affected group.

Data S1. (separate file)

Identification hit list for the polar metabolites in cortical bone samples measured by the HF-UPLC-IM-TOF-HRMS.

Data S2. (separate file)

Identification hit list for the polar metabolites in trabecular bone samples measured by the HF-UPLC-IM-TOF-HRMS.

Data S3. (separate file)

Identification hit list for the less-polar/apolar metabolites in cortical bone samples measured by the HF-UPLC-IM-TOF-HRMS.

Data S4. (separate file)

Identification hit list for the less-polar/apolar metabolites in trabecular bone samples measured by the HF-UPLC-IM-TOF-HRMS.

Data S5. (separate file)

Identification hit list for the less-polar/apolar metabolites in cortical bone samples measured by the nano-UHPLC-Orbitrap-HRMS.

Data S6. (separate file)

Identification hit list for the less-polar/apolar metabolites in trabecular bone samples measured by the nano-UHPLC-Orbitrap-HRMS.
